# Flow and On-Water Synthesis and Cancer Cell Cytotoxicity of Caffeic Acid Phenethyl Amide (CAPA) Derivatives

**DOI:** 10.3390/ijms25158051

**Published:** 2024-07-24

**Authors:** Anthony Saucedo, Muppidi Subbarao, Mauricio Jemal, Nakya L. Mesa-Diaz, Jadyn L. Smith, Alexandra Vernaza, Liqin Du, Sean M. Kerwin

**Affiliations:** 1 Department of Chemistry and Biochemistry, Texas State University, San Marcos, TX 78666, USA; atsau@umich.edu (A.S.); gnb37@txstate.edu (M.S.); l_d141@txstate.edu (L.D.); 2 Materials Science, Engineering, and Commercialization Program, Texas State University, San Marcos, TX 78666, USA; maj94@txstate.edu

**Keywords:** anticancer, natural product, neuroblastoma, Wittig reaction, flow synthesis, on-water synthesis

## Abstract

Caffeic acid phenethyl ester (CAPE) is a phenolic natural product with a wide range of biological activities, including anticancer activity; however, the ester group of CAPE is metabolically labile. The corresponding amide, CAPA, has improved metabolic stability but limited anticancer activity relative to CAPE. We report the synthesis using flow and on-water Wittig reaction approaches of five previously reported and five novel CAPA analogues. All of these analogues lack the reactive catechol functionality of CAPA and CAPE. Cytotoxicity studies of CAPE, CAPA, and these CAPA analogues in HeLa and BE(2)-C cells were carried out. Surprisingly, we found that CAPA is cytotoxic against the neuroblastoma BE(2)-C cell line (IC_50_ = 12 µM), in contrast to the weak activity of CAPA against HeLa cells (IC_50_ = 112 µM), and the literature reports of the absence of activity for CAPA against a variety of other cancer cell lines. One novel CAPA analogue, **3f**, was identified as having cytotoxic activity similar to CAPE in HeLa cells (IC_50_ = 63 µM for **3f** vs. 32 µM for CAPE), albeit with lower activity against BE(2)-C cells (IC_50_ = 91 µM) than CAPA. A different CAPA analogue, **3g**, was found to have similar effects against BE(2)-C cells (IC_50_ = 92 µM). These results show that CAPA is uniquely active against neuroblastoma cells and that specific CAPA analogues that are predicted to be more metabolically stable than CAPE can reproduce CAPA’s activity against neuroblastoma cells and CAPE’s activity against HeLa cells.

## 1. Introduction

**Figure 1:**
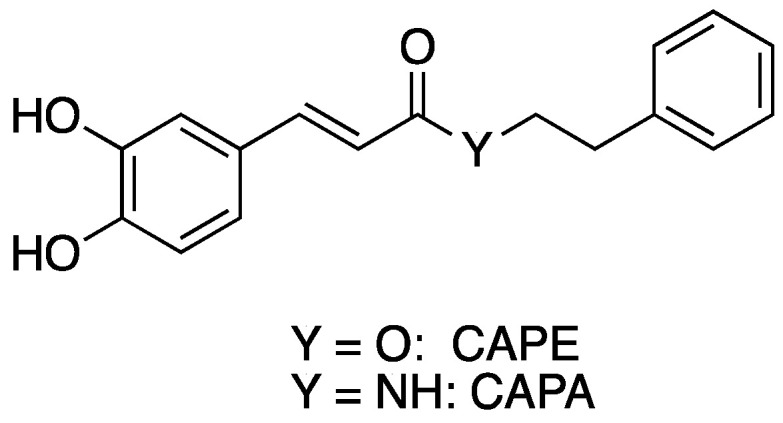
Structure of CAPE and CAPA.

Caffeic acid phenethyl ester (CAPE) (Figure 1) is a phenolic natural product isolated from bee propolis. Like propolis, CAPE has been reported to have a wide range of biological activity, including antimicrobial, anti-inflammatory, antioxidant, and immunostimulatory effects [1,2,3,4,5]. However, the metabolic liability of CAPE has led to the search for more metabolically stable analogues [6]. One promising approach to CAPE analogs with increased stability involves replacing the metabolically unstable ester linkage in CAPE with an amide moiety to give caffeic acid phenethyl amide (CAPA) (Figure 1). This change in going from CAPE to CAPA introduces “amidicity” [7], which has been shown to be a fundamental phenomenon and driving force controlling reactivity or functionality and can be measured and indexed. In some cases, the biological activity of CAPA mirrors that of CAPE. For example, CAPA is similar to CAPE in its reported cytoprotective activity against oxidative insult to human umbilical vein endothelial cells (HUVEC) [8]. CAPE and CAPA both are potent inhibitors of leukotriene biosynthesis in vitro [9], antifungal agents that synergize with fluconazole [10], and inhibitors of biofilm formation and quorum sensing [11]. However, CAPA does not possess all the biological activities associated with CAPE; the anti-cancer activity of CAPA is much lower than that of CAPE [12,13,14,15].

To address the issue of CAPA’s relative lack of anticancer activity compared to CAPE, we set out to explore analogues of CAPA that may recapture the anticancer properties of CAPE while possessing the increased metabolic stability associated with the ester-to-amide replacement. The mechanism of the anticancer activity of CAPE is not well established and likely varies from one cancer cell type to another [12,13,14,15]. The hydrolysis of CAPE to caffeic acid and 2-phenylethanol does not appear to be involved, as caffeic acid itself does not display cytotoxicity to cancer cells, and other esters of caffeic acid can have superior in vitro activity to CAPE [13,14,15]. Our focus here was on less readily hydrolyzed CAPA analogs with varied substituents on the caffeic acid catechol ring. Substituting the catechol moiety of CAPE or CAPA with alternative substituted phenyl groups has the added benefit of further increasing the metabolic stability of these analogs by avoiding the reactivity and extensive phase II metabolism associated with catechol groups.

There are a variety of synthetic approaches that have been used to prepare CAPE and CAPA analogues, but the most common include the ester or amide coupling of caffeic acid derivatives or the Wittig olefination of appropriate benzaldehydes [16]. Continuous flow methods offer several advantages to traditional batch methods for the synthesis of biologically active compounds in terms of safety, sustainability, and efficiency [17]. Specifically, Wittig olefination carried out in flow reactors has been used to prepare cinnamic ester derivatives [18] and cinnamides [19,20]. However, there have been no reports of flow reaction conditions to prepare CAPE and CAPA analogues. Wittig reactions have been carried out in water employing water-soluble phosphonium salts [21], but the advantages of “on-water” Wittig reactions of typical, water-insoluble partners have only recently been recognized [22,23]. While caffeic esters have been prepared on-water from preformed phosphorylides [24], there are no reports of this green approach to caffeic amides employing precursor phosphonium salts in aqueous base. Here, we report the flow and on-water synthesis of previously reported and novel CAPA analogues. We also report the unique and unexpected cytotoxicity of CAPA against BE(2)-C neuroblastoma cells and describe the cancer cell cytotoxicity of these CAPA analogues against HeLa adenocarcinoma and BE(2)-C cells in comparison to the activity of CAPE and CAPA.

## 2. Results

Amides are more stable than esters to hydrolysis, and the reactivity of specific amides can be related to the “amidicity” of these compounds, a quantitative measure of the degree to which the amide group is stabilized through conjugation [7]. Amidity is determined computationally by comparison of the enthalpy of hydrogen addition to the amide carbonyl relative to that of model systems defined as 100% (*N*,*N*-dimethylacetamide) or 0% (azaadamantane-2-one) amidity. Based on this method, we determined that CAPA has 115.8% amidity, indicating that the amide carbonyl group of CAPA is more resistant to attack than *N*,*N*-dimethylacetamide, which is reflective of a highly stable amide moiety.

**Scheme 1:**
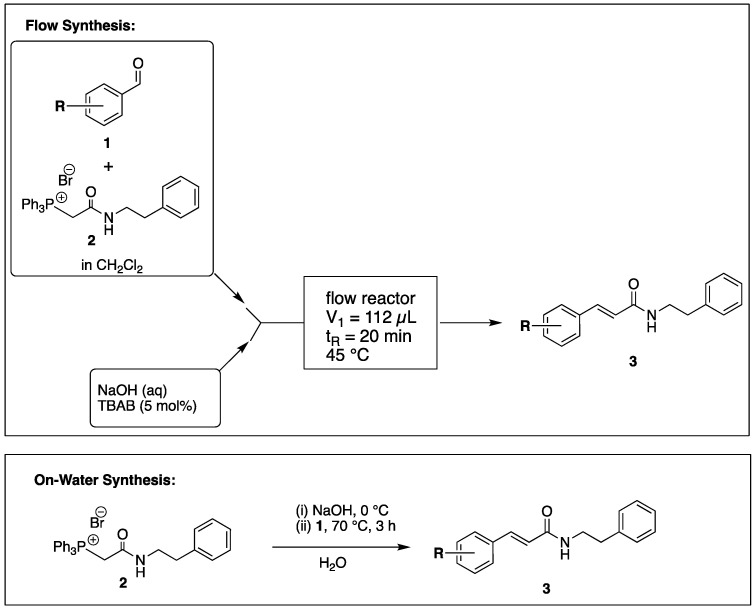
Procedure for Flow and On-Water Synthesis of CAPA Analogues.

The flow reactor design for the preparation of CAPA analogues is shown in Scheme 1. A glass microreactor with two inlets and channel dimensions of 700 µm width × 500 µm depth containing a mixing region and overall effective reaction volume of 112 µL (Figure S1) was heated to 45 °C. One inlet was connected to a syringe containing a solution of the substituted benzaldehyde (**1**) and (2-oxo-2-(phenethylamino)ethyl)triphenylphosphonium bromide (**2**) in CH_2_Cl_2_. The other inlet was connected to a syringe containing 160 mM NaOH in water containing 5 mol% of tetrabutylammonium bromide (TBAB). The flow from both syringes was equal and set to provide a residence time of 45 min. The outlet was plumbed to a collection flask, and after the run was complete, the product was isolated after liquid/liquid separation. Purification was carried out by flash chromatography.

Wittig reactions of benzaldehydes (**1**) and phosphonium bromide (**2**) on-water were carried out in water (0.8 M with respect to both **1** and **2**) with the addition of NaOH (4 equiv.) and heating at 70 °C for 3 h, followed by extraction of products into CH_2_Cl_2_ and purification by flash chromatography.

By employing different substituted benzaldehyde inputs (**1a**–**j**), ten different CAPA analogs (**3a**–**j**) were prepared, as shown in Table 1. The yields for the flow reactions were generally low, as most reactions failed to go to completion. Optimization efforts to improve the yields included increasing the equivalents of the phosphonium bromide reagent **2**, increasing the temperature, and increasing the residence time. Control reactions carried out in the absence of phase-transfer catalyst TBAB did not afford product. The yields reported for flow reactions represent the best conditions found and described in Table 1, which were limited by the temperature that could be attained and the maximum residence time attainable in the flow reactor. Reactions carried out on-water generally proceeded with higher, albeit moderate yields. Notably, both methods afforded >90% diastereomeric purity of the desired (*E*)-isomers, which is superior to some other methods plagued by the formation of mixtures of difficult-to-separate (*E*)- and (*Z*)-isomers [8].

With the CAPA analogues **3a**–**j** in-hand, we set out to determine if any of these display cytotoxicity against cancer cells that is superior to that of CAPA and perhaps even similar to that of CAPE itself. For these studies, we selected HeLa adenocarcinoma cells for our initial cytotoxicity screen, as the activity of CAPE in this cell type is well established [25,26]. Cytotoxicity was determined using the MTT assay [27]. As shown in Table 2, CAPE displays µM activity against HeLa cells, in accord with the literature data [25,26]. In contrast, CAPA has very low activity that is statistically different from that of CAPE (*p* < 0.05). While the CAPA analogues **3a**–**e** and **i**–**j** were inactive against HeLa cells, analogue **3g** displayed similar activity to CAPA, and analogue **3f** displayed activity that is superior to CAPA and close to that of CAPE. Analogue **3h** showed very low activity against HeLa cells.

We next examined the activity of CAPE, CAPA, and selected CAPA analogues against the neuroblastoma cell line BE(2)-C. Interestingly, while CAPE was more active against BE(2)-C than CAPA (*p* = 0.07), CAPA displayed markedly better activity against BE(2)-C than HeLa cells (*p* < 0.05) (Table 2). CAPA analogues **3a**, **3d**, **3i**, and **3j**, which were inactive against HeLa cells, were also inactive against the neuroblastoma cell line. CAPA analogues **3f** and **3g** displayed activity against both HeLa and BE(2)-C cells. While analogue **3f** was less active against BE(2)-C cells compared to HeLa cells, analogues **3g** and **3h** were similar to CAPA in being more active against the neuroblastoma cell line (see Table S1 for statistical analysis).

## 3. Discussion

CAPA has been shown to have no cytotoxicity against LNCaP human androgen-dependent prostate cancer cells [15], U-937 human lymphoma cells [28], or KMM-1 human myeloma cells [14]. Here, we show that CAPA is only weakly active against HeLa human adenocarcinoma cells (IC_50_ > 100 µM), as would be expected based upon the literature reports in other human cancer cell lines. However, we found that CAPA is active against human neuroblastoma BE(2)-C cells, with an IC_50_ of 21 µM. Given that CAPA is non-cytotoxic against normal human cells [8,29], the finding of anti-neuroblastoma activity for CAPA is significant, as selectivity for cancer cells is an important requirement for anticancer therapy.

A number of previously unreported CAPA analogs (**3d**–**h**) as well as the previously reported compounds **3a**–**c** and **3i,j** were prepared using Wittig flow and on-water chemistry. In general, the on-water method was found superior in terms of yields and has the advantage that no special equipment is required; however, both methods are operationally simple and provide a high degree of control of the stereochemistry of the product caffeic amides. CAPE, CAPA, and these analogues were assayed for cancer cell cytotoxicity against HeLa cells. One new compound, **3f**, with 4-fluoro-3-methoxy substituents, displayed cytotoxicity against HeLa cells that is superior to CAPA and about two times that of CAPE. This is remarkable in that the regioisomeric 3-fluoro-4-methoxy analogue **3e** was inactive against HeLa cells. While the 4-fluoro analogue **3j** and 3,4-difluoro analogue **3d** were also inactive against HeLa cells, the 2,4-difluoro compound **3g** displayed weak activity against HeLa cells, similar to CAPA. The 2-methoxy analogue **3h** showed only very weak activity against HeLa cells. Taken together, these results point to a tight geometric requirement for substituents about the ring corresponding to the catechol ring of CAPA for HeLa cell activity, with preference for 4-fluoro substituent in combination with substituents at the 2- or 3-positions.

CAPE, CAPA, and these analogues were also tested against the neuroblastoma cell line BE(2)-C. Surprisingly CAPA displayed activity in this cell line, although CAPE was more active (*p* < 0.05). This indicates that neuroblastoma may be a good target for CAPA and its analogues. CAPA analogues **3f**–**h** were also active against BE(2)-C cells, although none of these had activity superior to that of CAPA. Interestingly, while **3f** was the most active of the analogues examined here against HeLa cells, **3g** and **3f** had similar activity against BE(2)-C cells. This may indicate a similar but distinct structure–activity relationship for CAPA analogues in these two cancer cell lines.

## 4. Materials and Methods

Unless otherwise noted, all materials were obtained from commercial suppliers and were used without further purification: CAPE (>98%) from CaymanChemical Co., Ann Arbor, MI, USA; CAPA (98%) from TargetMol, Boston, MA, USA; 4-fluorobenzaldehyde (98%), 3,4-dimethoxybenzaldehyde (99%) from Acros, Fair Lawn, NJ, USA; 3,4-difluorobenzaldehyde (95%) and 4-fluoro-3-methoxybenzaldehyde (97%) from Matrix Scientific, Columbia, SC, USA; 3-fluoro-4-methoxybenzaldehyde (99%), piperonal (99%), *p*-anisaldehyde (99%), and 3,4,5-trimethoxybenzaldehyde (98%) from Millipore Sigma, St. Louis Missouri, MI, USA; and 2,4-difluorobenzaldehyde (98%) from TCI, Houston, TX, USA. (2-Oxo-2-(phenethylamino)ethyl)triphenylphosphonium bromide (**2**) was prepared by the literature method [8]. Flash chromatography was performed on a Teledyne CombiFlash with RediSep Rf silica gel (230–400 mesh) using the mobile phase indicated. Melting points (open capillary) were determined on a Electrothermal series IA 9000 digital melting point apparatus and are uncorrected. Unless otherwise noted, ^1^H and ^13^C NMR spectra were obtained on a Bruker Advance Spectrometers operating at 400 MHz (100 MHz for ^13^C) or 500 MHz (126 MHz for ^13^C) and were determined in CDCl_3_. Chemical shifts are reported in ppm using solvent as internal standard (7.26 ppm for ^1^H and 77.0 ppm for ^13^C in CDCl_3_). Low-resolution mass spectra were obtained on a Advion ExpressionL compact mass spectrometer with atmospheric pressure chemical ionization (APCI) or electrospray ionization (ESI). High-resolution mass spectra were obtained on a Waters SynaptXS spectrometer with ESI or and Agilent 6530 Q-TOF LC/MS with chemical ionization (CI). Copies of all NMR and MS spectra are available in the Supporting Information. Flow chemistry was carried out on a Future Chemistry FlowStart Evo system with a M-111 microreactor.

General Procedure 1—Flow Wittig Reaction: (*E*)-phenethyl 3-(3,4,5-trimethoxyphenyl)acrylamide (**3b**): A syringe was charged with a solution of 15.7 mg (0.08 mmol) of 3,4,5-trimethoxybenzaldehyde and 73.59 mg (0.16 mmol) of (2-oxo-2-(phenethylamino)ethyl)triphenylphosphonium bromide (**2**) in 1 mL of CH_2_Cl_2_. A second syringe was charged with a solution of 1.29 mg (5 mol%) TBAB and 6.4 mg (0.16 mmol) NaOH in 1 mL in H_2_O. The flow reactor was set to run with equal flow from both syringes and a residence time of 20 min at 45 °C. The resultant mixture was then transferred to a 30 mL sep. funnel, and the aqueous layer was washed with CH_2_Cl_2_. (3 × 3 mL), and then, the organic layer was dried over Na_2_SO_4_, filtered, and concentrated under reduced pressured to give a slightly yellow oil that was subjected to flash chromatography (55%/45% EtOAc:Hex with 0.1% triethylamine) to afford 14.8 mg (52% yield) of compound **3b** as a white solid after chromatography: ^1^H NMR (400 MHz, CDCl_3_) δ: 7.52 (1H, d, *J* = 15.5 Hz, CH=CH), 7.35–7.31 (2H, m, ArH), 7.23 (3H, m, ArH), 6.70 (2H, s, ArH), 6.23 (1H, d, *J* = 15.5 Hz, CH=CH), 5.66–5.64 (1H, m, NH), 3.86 (6H, s, -OCH_3_), 3.86 (3H, s, -OCH_3_), 3.67 (1H, q, *J* = 6.4 Hz, CH_2_CH_2_), 2.89 (2H, t, *J* = 6.8 Hz, CH_2_CH_2_); ^13^C NMR (100 MHz, CDCl_3_) δ: 165.8, 153.4, 141.0, 139,6, 138,.8, 130.3, 128.8, 128,7, 126.5, 119.9, 104.9, 60.9, 56.1, 40.7, 35.6; APCI MS *m*/*z*: 342 (MH^+^). Matches lit [30].

(*E*)-phenethyl 3-(3,4-dimethoxyphenyl)acrylamide (**3a**): Following the general procedure 1 starting with 3,4-dimethoxybenzaldehyde afforded 27 mg (24% yield) of a white solid after flash chromatography: mp = 131–132 °C (lit. 126–128 °C); ^1^H NMR (400 MHz, CDCl_3_) δ: 7.55 (1H, d, *J* = 15.5 Hz, CH=CH), 7.31–7.27 (2H, m, ArH), 7.23–7.19 (3H, m, ArH), 7.04 (1H, dd, *J* = 8.3, 1.8 Hz, ArH), 6.98 (1H, d, *J* = 1.8 Hz, ArH), 6.80 (1H, d, *J* = 8.3 Hz, ArH), 6.25 (1H, d, *J* = 15.5 Hz, CH=CH), 6.01 (1H, s, -OCH_3_), 3.86 (3H, s, -OCH_3_), 3.84 (3H, s, -OCH_3_), 3.64 (2H, q, *J* = 5.9 Hz, CH_2_CH_2_), 2.87 (2H, t, *J* = 6.9 Hz, CH_2_CH_2_); ^13^C NMR (100 MHz, CDCl_3_) δ: 166.2, 150.5, 149.0, 140.7, 138.8, 128.7, 128.6, 127.7, 126.3, 121.8, 118.4, 111.0, 109.6, 55.8, 55.7, 40.7. 35.6; APCI MS *m*/*z*: 312 (MH^+^). Matches lit [30].

(*E*)-phenethyl 3-(4-methoxyphenyl)acrylamide (**3c**): Following the general procedure 1 starting with 4-methoxybenzaldehyde afforded 13.6 mg (12% yield) of a white solid after flash chromatography (60% EtOAc/hex): ^1^H NMR (400 MHz, CDCl_3_) δ: 7.57 (1H, d, *J* = 15.6 Hz, CH=CH), 7.43–7.42 (2H, m, ArH), 7.34–7.31 (2H, m, ArH), 7.24–7.22 (3H, m, ArH), 6.88–6.87 (2H, m, ArH), 6.19 (1H, d, *J* = 15.6 Hz, CH_2_CH_2_), 5.59 (1H, s(br), NH), 3.82 (3H, s, -OCH_3_), 3.66 (2H, q, *J* = 6.4 Hz, CH_2_CH_2_), 2.89 (2H, t, *J* = 6.9 Hz, CH_2_CH_2_). ^13^C NMR (100 MHz) δ: 166.2, 160.9, 140.7, 138.9, 129.3, 128.8, 128.7, 127.5, 126.5, 118.2, 114.3, 55.3, 40.7, 35.7; APCI MS *m*/*z*: 282 (MH^+^). Matches lit [30].

(*E*)-phenethyl 3-(3,4-difluorophenyl)acrylamide (**3d**): Following the general procedure 1 starting with 3,4-difluorobenzaldehyde afforded 13.2 mg (14% yield) of a white solid after flash chromatography (15–40% EtOAc/hex): mp 143–145 °C; IR 3303, 11,656, 1622, 1550, 1518, 1275, 1197, 966 cm^−1^; ^1^H NMR (400 MHz, CDCl_3_) δ: 7.51 (1H, d, *J* = 15.5 Hz, CH=CH), 7.34–7.29 (2H, m, ArH), 7.28–7.11 (6H, m, ArH), 6.25 (1H, d, *J* = 15.5 Hz, CH=CH), 5.78 (1H, s(br), NH), 3.66 (2H, q, *J* = 6.4 Hz, CH_2_CH_2_), 2.89 (2H, t, J = 6.4 Hz, CH_2_CH_2_); ^13^C NMR (100 MHz, CDCl_3_) δ: 165.2, 150.9 (dd, *J*_C-F_ = 252.4, 13.1 Hz), 150.5 (dd, *J*_C-F_ = 249.1, 12.9 Hz), 138.8, 138.7, 132.0 (dd, *J*_C-F_ = 6.1, 4.1 Hz), 128.7 (d, *J*_C-F_ = 4.7 Hz), 126.6, 124.5 (dd, *J*_C-F_ = 6.4, 3.4 Hz), 121.7, 121.6, 117.6 (d, *J*_C-F_ = 17.7 Hz), 115.8 (d, *J*_C-F_ = 17.6 Hz), 40.8, 35.6; CI MS *m*/*z*: 288.1 (MH^+^); HR CIMS calcd. for C_17_H_16_NOF_2_ 288.1200 (MH^+^), found 288.1200.

(*E*)-phenethyl 3-(3-fluoro-4-methoxyphenyl)acrylamide (**3e**): Following the general procedure 1 starting with 3-fluoro-4-methoxybenzaldehyde afforded 44 mg (37% yield) of a white solid after flash chromatography (40% EtOAc/hex): mp 152–152.5 °C; IR 3297, 3078, 3029, 29,665, 1657, 1613, 1577, 1547, 1519, 1270, 1119 cm^−1^; ^1^H NMR (400 MHz, CDCl_3_) δ: 7.51 (1H, d, *J* = 15.5 Hz, CH=CH), 7.32–7.28 (1H, m, ArH), 7.24–7.14 (5H, m, ArH), 6.88 (1H, t, *J* = 8.5 Hz, ArH), 6.23 (1H, d, *J* = 15.5 Hz, CH=CH), 6.03 (1H, s(br), NH), 3.88 (3H, s, -OCH_3_), 3.64 (2H, q, *J* = 6.3 Hz, CH_2_CH_2_), 2.88 (2H, t, *J* = 7.0 Hz, CH_2_CH_2_). ^13^C NMR (100 MHz) δ: 165.8, 152.2 (d, *J*_C-F_ = 246.7 Hz), 148.8 (d, *J*_C-F_ = 11.0 Hz), 139.5 (d, *J*_C-F_ = 2.4 Hz), 138.7, 128.7, 128.5, 128.1 (d, *J*_C-F_ 6.6 Hz), 126.5, 125.0 (d, *J*_C-F_ = 3.1 Hz), 119.6, 114.1 (d, *J*_C-F_ = 18.9 Hz), 113.1 (d, *J*_C-F_ = 2.1 Hz), 56.1, 40.8, 35.6. CIMS *m*/*z* 300 (MH^+^); HR CIMS calcd. for C_18_H_20_FNO_2_ (MH^+^) 300.1400, found 300.1398.

(*E*)-phenethyl 3-(4-fluoro-3-methoxyphenyl) acrylamide (**3f**). Following the general procedure 1 starting with 4-fluoro-3-methoxybenzaldehyde afforded 40 mg (33% yield) of a white needles after flash chromatography (40% EtOAc/hex). Follow the general procedure 2 starting with 62 mg of aldehyde afforded 59 mg (49% yield) after flash chromatography: mp = 119–120 °C; IR (ATR, neat): 3297, 3071, 3029, 2946, 1739, 1654, 1621, 1603, 1546, 1519, 1467, 1410, 1374, 1319, 1299, 1283, 1261, 1209, 1191, 1162, 1118, 1045, 1029 cm^−1^; ^1^H NMR (400 MHz, CD_3_COCD_3_) δ: 7.49 (1H, d, *J* = 15.6 Hz, CH=CH), 7.39 (1H, s(br), NH), 7.33–7.30 (1H, m, ArH), 7.29–7.23 (5H, m, ArH), 7.21–7.18 (2H, m, ArH), 6.63 (1H, dd, *J* = 15.6, 0.5 Hz, CH=CH), 3.91 (3H, s, -OCH_3_), 2.86 (2H, t, *J* = 7.4 Hz, CH_2_CH_2_); ^13^C NMR (100 MHz, CD_3_COCD_3_) δ: 165.9, 153.7 (d, *J*_C-F_ = 248.0 Hz), 148.8 (d, *J*_C-F_ = 11.0 Hz), 140.5, 139.2 133.2 (d, *J*_C-F_ = 3.9 Hz), 129.2, 127.0, 122.9 (d, *J*_C-F_ = 2.3 Hz), 121.4, 121.3, 116.9 (d, *J*_C-F_ = 18.5 Hz), 113.3 (d, *J*_C-F_ = 2.1 Hz), 56.4, 41.6, 36.5. CIMS *m/z:* 300 (MH^+^); HR CIMS calcd. for C_18_H_20_FNO_2_ (MH^+^) 300.1400, found 300.1402.

General Procedure 2—On-Water Wittig Reaction: (*E*)-3-(2,4-difluorophenyl)-*N*-phenethylacrylamide (**3g**): To a stirred solution of phosphonium salt (201 mg, 0.4 mmol) in 2 mL of water at 0 °C, sodium hydroxide (64 mg, 1.6 mmol) portion wise added and followed by addition of 2,4-difluorobenzaldehyde (56.8 mg, 0.4 mmol). The resulting mixture was stirred at 70 °C for 3 h. After completion of the reaction (monitored by TLC), the reaction mixture was diluted with water (10 mL) and the aqueous layer was extracted with CH_2_Cl_2_ (2 × 25 mL). The combined organic layers were washed with brine solution (20 mL), dried over sodium sulphate, and concentrated under reduced pressure. Purification by flash chromatography (40% EtOAc/hex) followed by recrystallisation from CH_2_Cl_2_ and hexane afforded 59 mg (52% yield) of compound **3g** as a white solid: mp = 131–133 °C; IR 3393, 1670, 1616, 1550, 1521, 1472, 1167 cm^−1^; ^1^H NMR (400 MHz, CDCl_3_) *δ*: 7.66 (1H, d, *J* = 15.7 Hz, CH=CH), 7.47 (1H, q, *J* = 8.4 Hz, ArH), 7.38–7.31 (2H, m, ArH), 7.29–7.25 (3H, m, *J* = 6.7 Hz, ArH), 6.92–6.84 (2H, m, ArH), 6.42 (1H, d, *J* = 15.6 Hz, CH=CH), 5.68 (1H, s(br), NH), 3.69 (2H, q, *J* = 6.7 Hz, CH_2_CH_2_), 2.92 (2H, t, *J* = 6.9 Hz, CH_2_CH_2_); ^13^C NMR (126 MHz, CDCl_3_) δ: 165.8, 163.4 (dd, *J*_C-F_ = 252.7, 12.4 Hz), 161.5 (dd, *J*_C-F_ = 256.0, 12.1 Hz), 138.8, 133.0, 130.6 (dd, *J*_C-F_ = 9.5, 4.5 Hz), 128.8, 128.7, 126.6, 123.2 (d, *J*_C-F_ = 6.4 Hz), 119.4 (dd, *J*_C-F_ = 11.2, 3.2 Hz), 111.9 (dd, *J*_C-F_ = 21.7, 2.5 Hz), 104.6 (t, *J*_C-F_ = 25.7 Hz), 40.9, 35.7; ^19^F NMR (470 MHz, CDCl_3_) δ −107.25, −109.59; APCI MS *m*/*z*: 288.1 (MH^+^); HR CIMS calcd. for C_17_H_16_F_2_NO (MH^+^) 288.1194, found 288.1201.

(*E*)-3-(2-methoxyphenyl)-N-phenethylacrylamide (**3h**): Following the general procedure 2 starting with 2-methoxy benzaldehyde (54 mg) afforded 46 mg (41% yield) of white needles after flash chromatography (EtOAc/hex): mp = 131–133 °C; IR 3248, 1670, 1600, 1568, 1548, 1472, 1167 cm^−1^; ^1^H NMR (400 MHz, CDCl_3_) δ: 7.61 (1H, d, *J* = 15.6 Hz, CH=CH), 7.40–7.32 (2H, m, ArH), 7.30 (1H, dd, *J* = 6.3, 4.7 Hz, ArH), 7.28–7.23 (3H, m, ArH), 7.10 (1H, d, *J* = 7.7 Hz, ArH), 7.05–6.99 (1H, m, ArH), 6.92 (1H, dd, *J* = 8.2, 1.9 Hz, ArH), 6.33 (1H, d, *J* = 15.6 Hz, CH=CH), 5.68 (1H, s(br), NH), 3.84 (3H, s, -OCH_3_), 3.69 (2H, q, *J* = 6.7 Hz, 2H), 2.92 (2H, t, *J* = 6.9 Hz, 2H); ^13^C NMR (100 MHz, CDCl_3_) δ: 165.8, 159.8, 141.0, 138.9, 136.2, 129.8, 128.8, 128.7, 126.6, 120.9, 120.4, 115.4, 112.9, 55.3, 40.8, 35.7; APCI MS *m*/*z*: 282.1 (MH^+^); HR CIMS calcd. for C_18_H_20_NO_2_ (MH^+^) 282.1489, found 282.1505.

(*E*)-3-(benzo[*d*][1,3]dioxol-5-yl)-*N*-phenethylacrylamide (**3i**): Following the general procedure 2 starting with benzo[*d*][1,3]dioxole-5-carbaldehyde (60 mg) afforded 78 mg (66% yield) of a white solid after flash chromatography (EtOAc/hex): mp = 138–140 °C (lit. 133–136 °C); ^1^H NMR (400 MHz, CDCl_3_) δ: 7.56 (1H, d, *J* = 15.4 Hz, CH=CH), 7.35 (2H, t, *J* = 7.4 Hz, ArH), 7.26 (3H, dd, *J* = 13.8, 7.2 Hz, ArH), 6.99 (2H, d, *J* = 8.4 Hz, ArH), 6.80 (1H, d, *J* = 7.8 Hz, ArH), 6.19 (1H, d, *J* = 15.4 Hz, CH=CH), 5.99 (2H, s, OCH_2_O-), 5.74 (1H, s(br), NH), 3.68 (2H, q, *J* = 4.6 Hz, CH_2_CH_2_), 2.91 (2H, t, *J* = 6.8 Hz, CH_2_CH_2_); ^13^C NMR (126 MHz, CDCl_3_) δ: 166.1, 149.1, 148.2, 140.9, 138.9, 129.2, 128.8, 128.7, 126.6, 123.9, 118.6, 108.5, 106.4, 101.4, 40.8, 35.7; APCI MS *m*/*z*: 296.1 (MH^+^). Matches lit [30].

(E)-3-(4-fluorophenyl)-N-phenethylacrylamide (**3j**): Following the general procedure 2 starting with 4-fluorobenzaldehyde (50 mg) afforded 59 mg (55% yield) of a white solid after flash chromatography (EtOAc/hex): mp = 151–153 °C; ^1^H NMR (500 MHz, CDCl_3_) δ: 7.61 (1H, d, *J* = 15.4 Hz, CH=CH), 7.49 (2H, m, ArH), 7.36 (2H, m, ArH), 7.27 (3H, m, ArH), 7.07 (2H, t, *J* = 8.4 Hz, ArH), 6.27 (1H, d, *J* = 15.4 Hz, CH=CH), 5.64 (1H, s(br), NH), 3.70 (2H, q, *J* = 4.8 Hz, CH_2_CH_2_), 2.92 (2H, t, *J* = 6.5 Hz, CH_2_CH_2_); ^13^C NMR (126 MHz, CDCl_3_) δ: 165.8, 163.5 (d, *J*_C-F_ = 250.3 Hz), 139.8, 138.9, 131.1 (d, *J*_C-F_ = 2.5 Hz), 129.6 (d, *J*_C-F_ = 8.3 Hz), 128.8, 128.7, 126.6, 120.4, 115.9 (d, *J*_C-F_ = 21.9), 40.8, 35.7; ^19^F NMR (470 MHz, CDCl_3_) δ: −110.72; APCI MS *m*/*z*: 270.1 (MH^+^). Matches lit [31].

Computational Studies: All computational studies were carried out with the Gaussian16 [32] package. Geometry optimization and subsequent frequency calculations were carried out at the B3LYP/6-31G(d,p) level. The enthalpy of reaction for the addition of hydrogen to CAPA was determined from thermodynamic values calculated at 298 K. The resulting enthalpy of reaction was fit to Equation (1) in order to determine the amidity of CAPA.

Percent Amidity = [1.258 × ΔH (kJ/mol)] + 56.126(1)

Cytotoxicity Assays: HeLa cells were maintained in RPMI culture medium supplemented with 10% Equafetal bovine serum (EFBS) and 1% penicillin-streptomycin (P/S). Neuroblastoma BE(2)-C cells were maintained in DMEM/F12 culture medium supplemented with 10% EFBS and 1% P/S. For measuring the dose-dependent effect of a compound on cell viability, cells were plated into a 96-well plate and treated with a series of dilutions of individual compounds in four replicates for 4 days. After 4 days, cell viability was determined by MTT (3-(4,5-dimethylthiazol-2-yl)-2,5-diphenyltetrazolium bromide) assay [27]. Dose-dependent graphs were generated, and IC_50_ values were determined using the GraphPad Prism 9 software. The IC_50_ values correspond to the concentrations of the compounds that reduced the cell viability by 50% in comparison to the carrier (DMSO)-treated control condition. The mean and standard deviation (SD) of IC_50_ for each compound in each cell line were calculated from IC_50_’s obtained from at least two independent experiments. Statistical analysis was carried out by two-tailed *t*-test (*p* < 0.050) to determine non-equivalence of IC_50_ values.

## 5. Conclusions

The results reported here are significant in several ways. First, the discovery of CAPA’s activity against neuroblastoma cells is remarkable given the lack of activity against other human cancer cell types. The lack of cytotoxicity of CAPA in normal human cells indicates that CAPA may be a highly selective agent for neuroblastoma. The amidity of CAPA indicates that it is more metabolically stable to hydrolysis as compared to CAPE, which is a positive attribute for further exploration of CAPA and its analogues as a potential therapy for neuroblastoma.

Several CAPA analogues were prepared using both flow and on-water Wittig couplings. In general, the on-water method was found to be superior, as it does not require special equipment, provides higher yields, and is environmentally friendly. While CAPA is only weakly active against HeLa cells, it is significant that one of these analogues, **3f**, displays cytotoxicity against HeLa cells that is similar to that of CAPE. This shows that replacement of the catechol ring of CAPA can afford compounds that recapture the broad anticancer activity that is lost going from CAPE to CAPA while having the additional benefit of avoiding the reactivity of this catechol group. The lack of activity of the regioisomeric CAPA analogue **3e** indicates that there is a significant structure–activity relationship (SAR) for cytotoxicity against HeLa cells in these CAPA analogues. It is also notable that **3g** and **3f** are equipotent against BE(2)-C neuroblastoma cells, albeit less active than CAPA itself. This indicates a distinct SAR for BE(2)-C cell cytotoxicity for CAPA analogues compared to HeLa cell activity.

There is an interest in metabolically stable CAPE analogues that incorporate an amide isostere, as in CAPA, that can overcome the limited cancer cell cytotoxicity of CAPA. The anti-neuroblastoma activity of CAPA and analogues **3f** and **3g** reported here provides a starting point for future studies on the mechanism of this selective anti-cancer activity. In addition, the identification of the HeLa cell-active CAPA analogue **3f** demonstrates that judicious modification of the CAPA structure can lead to enhanced activity even in cancer cell lines in which CAPA itself is inactive. The work reported here focused on improvements of the catechol group of CAPA; future work that builds from the identification of **3f** and **3g** as anticancer CAPA analogues will focus on further optimization of other structural features.

## Figures and Tables

**Table 1:** Synthesis of CAPA Analogues.

Aldehyde (1)	Product (3)	Method	Yield
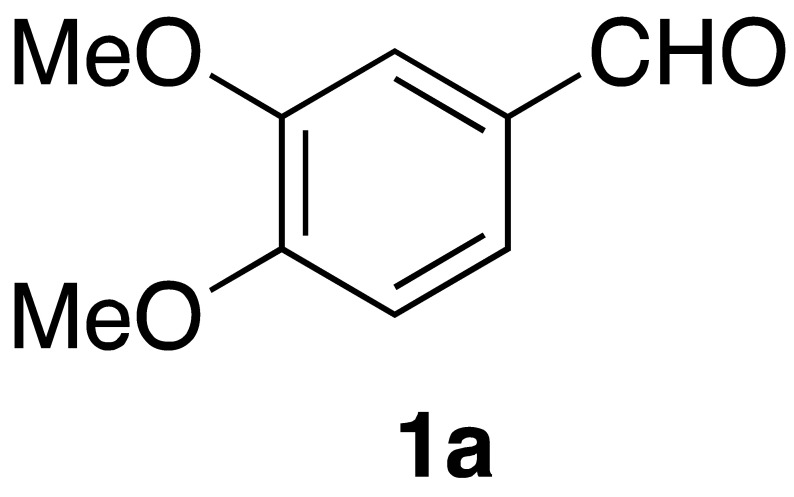	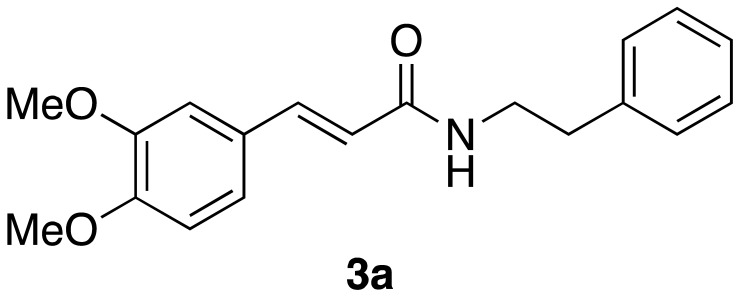	Flow	24%
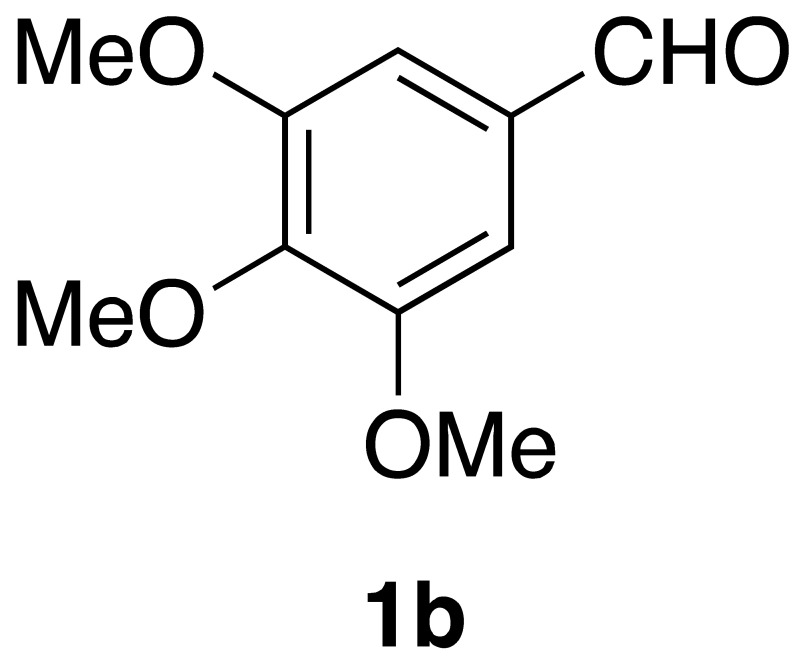	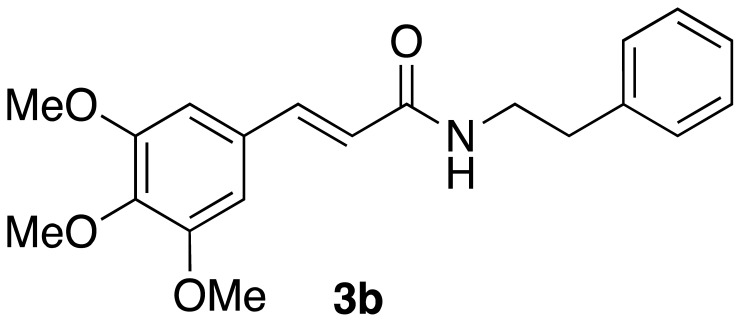	Flow	52%
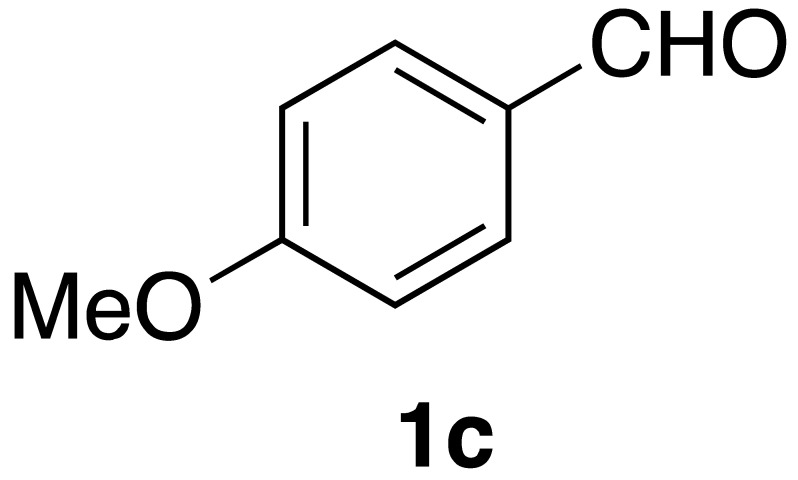	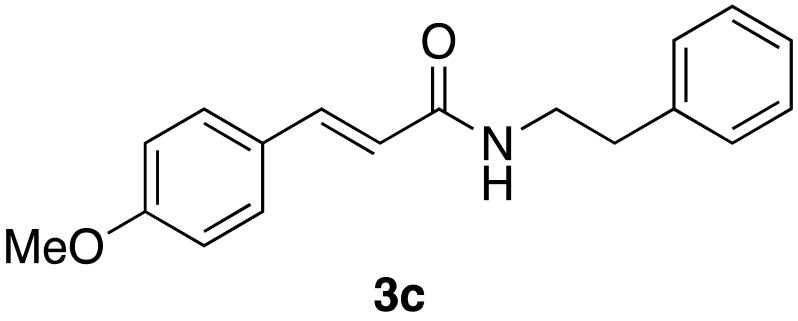	Flow	12%
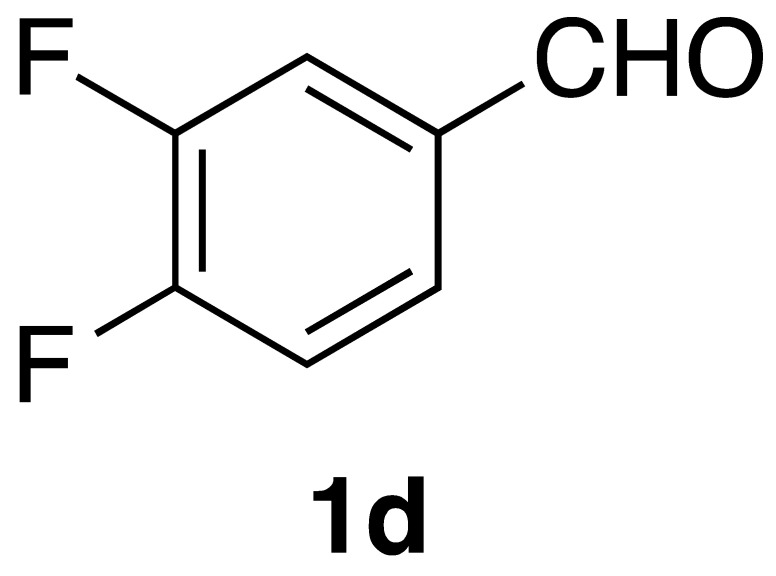	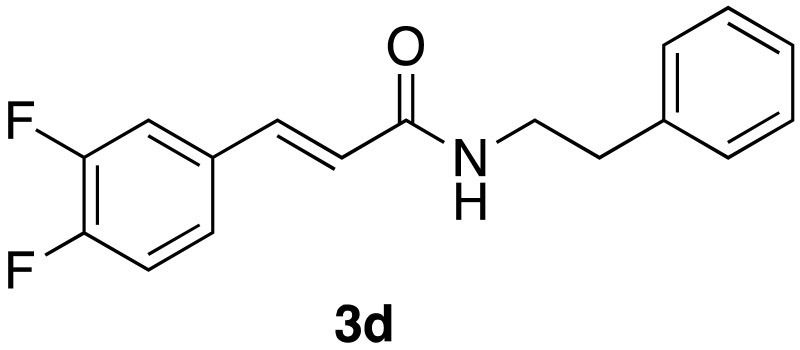	Flow	14%
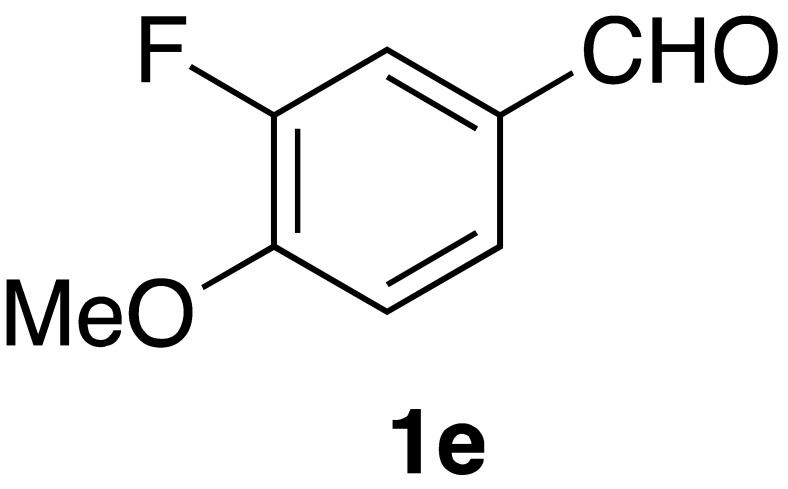	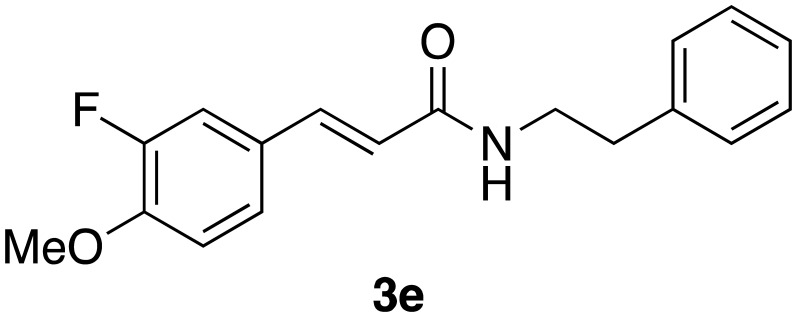	Flow	37%
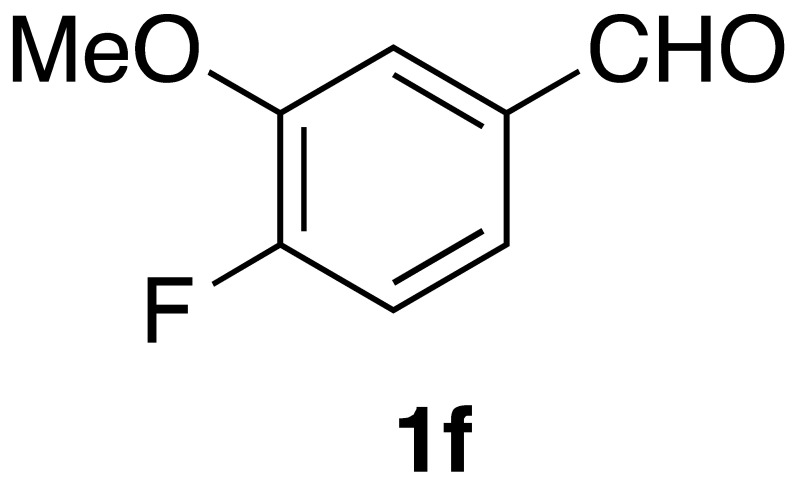	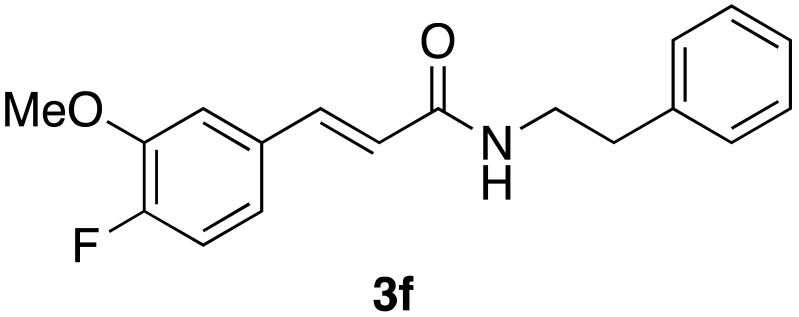	Flow On-water	33% 49%
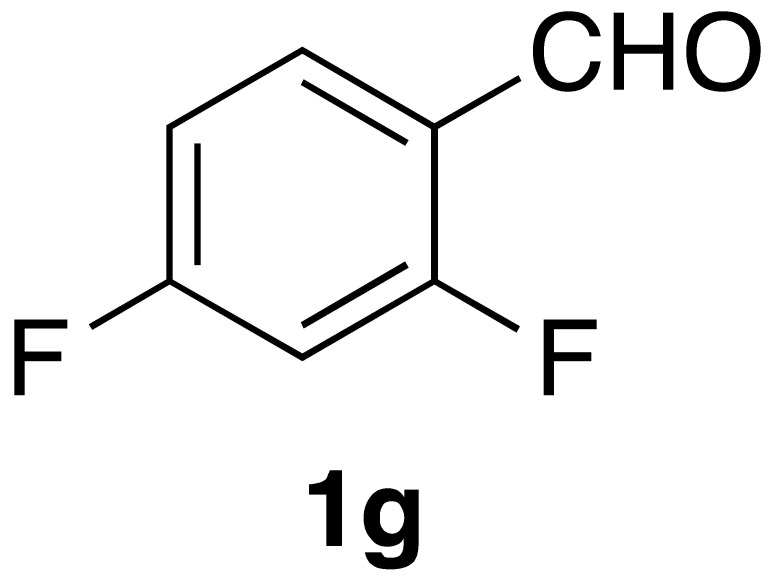	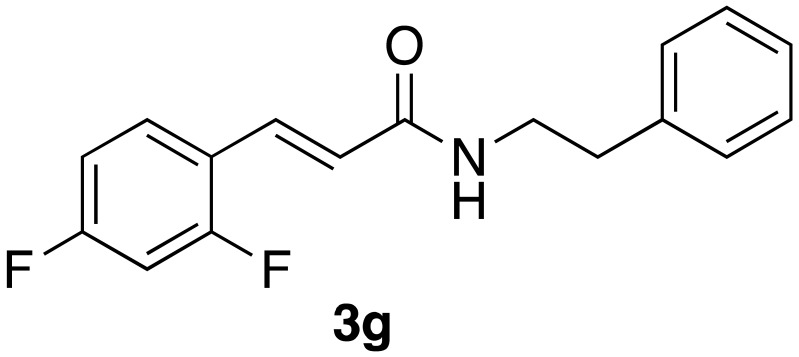	On-water	52%
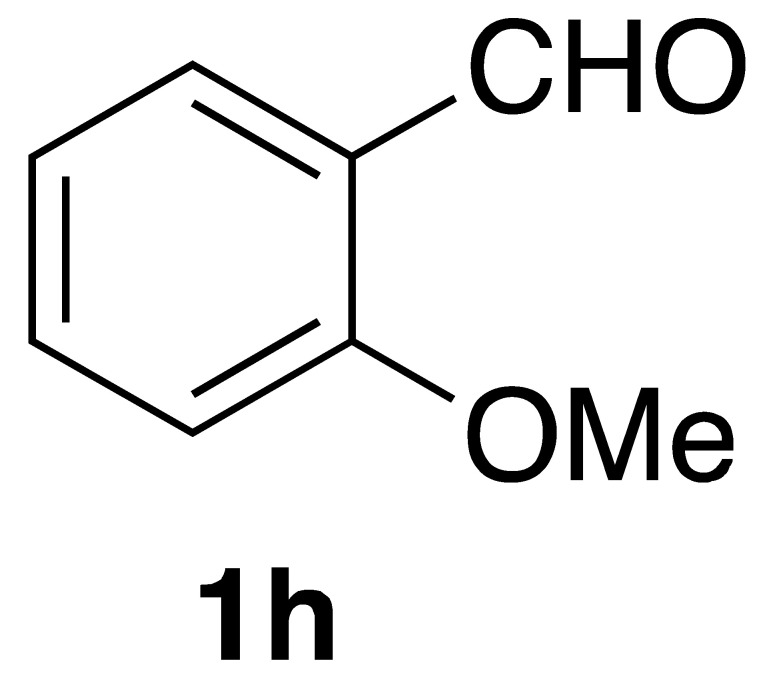	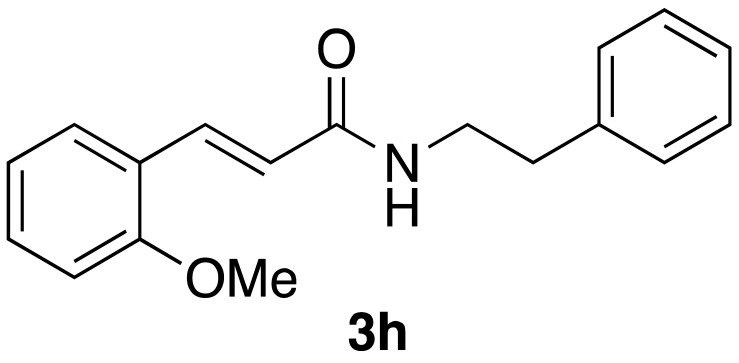	On-water	41%
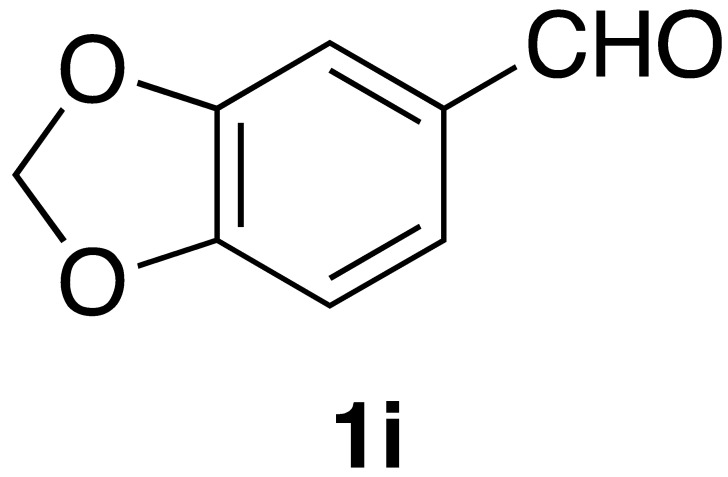	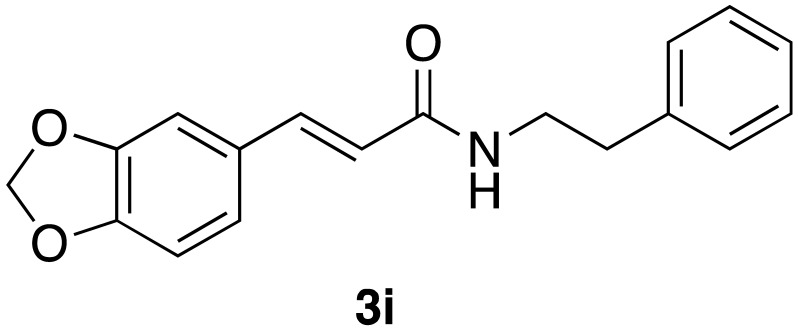	On-water	66%
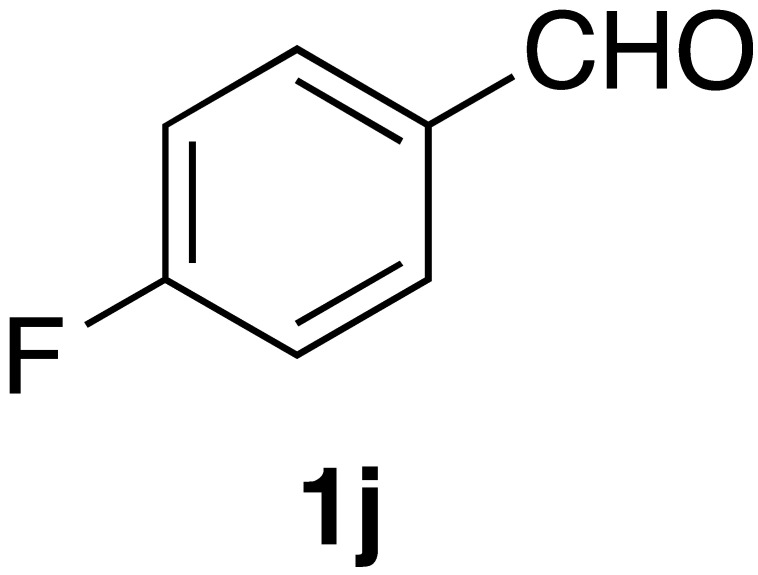	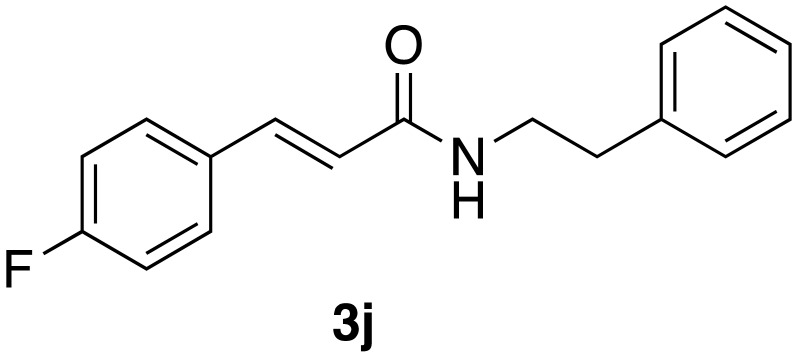	On-water	55%

**Table 2:** Cytotoxicity of CAPE, CAPA, and CAPA Analogues.

Compound	HeLa IC_50_ (µM)	BE(2)-C IC_50_ (µM)
CAPE	32 ± 12	5 ± 2
CAPA	112 ± 17	21 ± 2
**3a**	>600	>600
**3b**	>600	nd ^1^
**3c**	>600	nd
**3d**	>600	>600
**3e**	>600	nd
**3f**	63 ± 29	91 ± 52
**3g**	140 ± 58	92 ± 48
**3h**	600 ± 66	163.8 ± 0.6
**3i**	>600	>600
**3j**	>600	>600

^1^ not determined.

## Data Availability

The data presented in this study are available in the Supplementary Materials.

## Supplementary Materials

The following supporting information can be downloaded at: https://www.mdpi.com/article/10.3390/ijms25158051/s1.
