# Supplementary material for: Flow and On-Water Synthesis and Cancer Cell Cytotoxicity of Caffeic Acid Phenethyl Amide (CAPA) Derivatives
Source: Int J Mol Sci. 2024 Jul 24;25(15):8051. doi: 10.3390/ijms25158051 (PMC11312412; doi:10.3390/ijms25158051)
Supplement: Supplementary file 1 [file ijms-25-08051-s001.zip › ijms-3099590-supplementary.pdf]

**Supporting Information**

# Flow and On-Water Synthesis and Cancer Cell Cytotoxicity of Caffeic Acid Phenethyl Amide (CAPA) Derivatives

**Anthony Saucedo[1], Muppidi Subbarao[1], Mauricio Jemal[2], Nakya L. Mesa-Diaz[1], Jadyn L. Smith[1], Alexandra Vernaza[1], Liqin Du[1], and Sean M. Kerwin[1,2*]**

[1] Department of Chemistry and Biochemistry, Texas State University, San Marcos, TX, USA
[2] Materials Science, Engineering, and Commercialization Program, Texas State University, San Marcos, TX, USA
* Correspondence: smk89@txstate.edu

## Contents

Schematic of Flow Reactor (Figure S1) ……………………………………S2
$^{1}$H and $^{13}$C NMR Spectra for Compounds **3a-j**………………………………S3-S15
Cell Viability Curves of CAPE, CAPA, and **3a-j** against HeLa Cells………S16-S19
Cell Viability Curves of CAPE, CAPA, and **3a,d-f** against BE(2)-C Cells….S20-S23
Statistical Analysis of $IC_{50}$ values (Table S1)…………………….……………S24
Calculated Structures and Energies for CAPA and H2-CAPA…………..…S25-S26

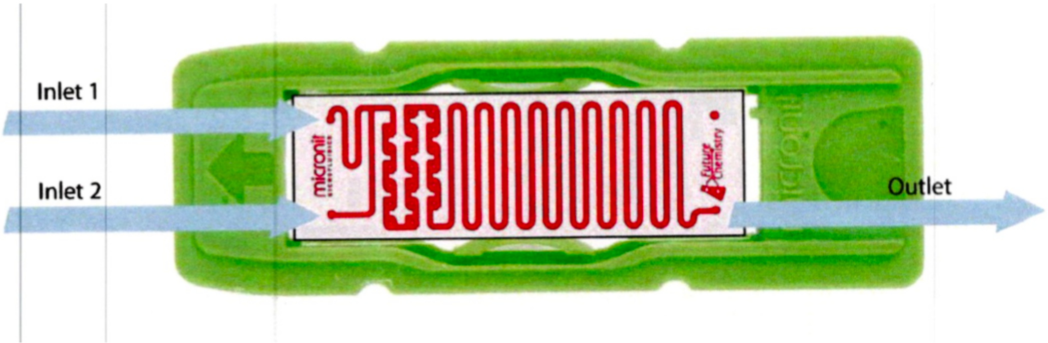


**Figure S1.** Layout of Future Chemistry microreactor used

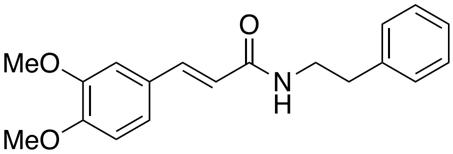

MeO
MeO
O
N
H


**3a**

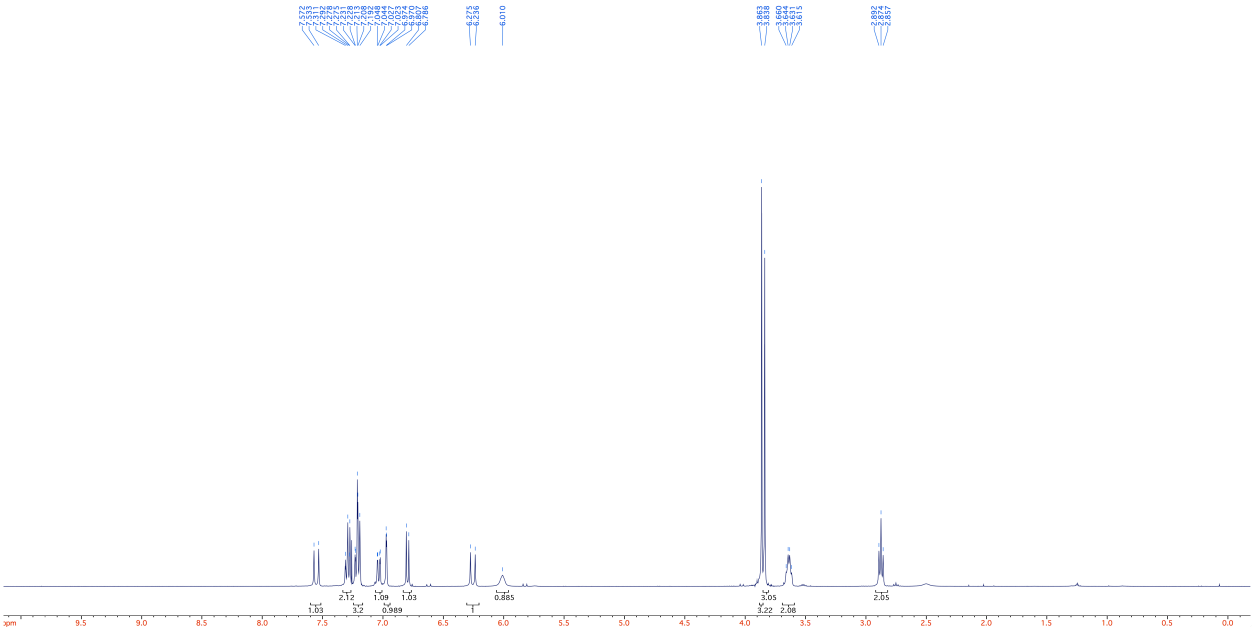

7.572
7.533
7.311
7.292
7.278
7.275
7.231
7.228
7.213
7.192
7.048
7.044
7.027
7.023
6.974
6.970
6.807
6.786
6.275
6.236
6.010
3.863
3.838
3.660
3.644
3.631
3.615
2.892
2.874
2.857
1.03
2.12
3.2
1.09
0.989
1.03
1
0.885
3.05
3.22
2.08
2.05
ppm
9.5
9.0
8.5
8.0
7.5
7.0
6.5
6.0
5.5
5.0
4.5
4.0
3.5
3.0
2.5
2.0
1.5
1.0
0.5
0.0


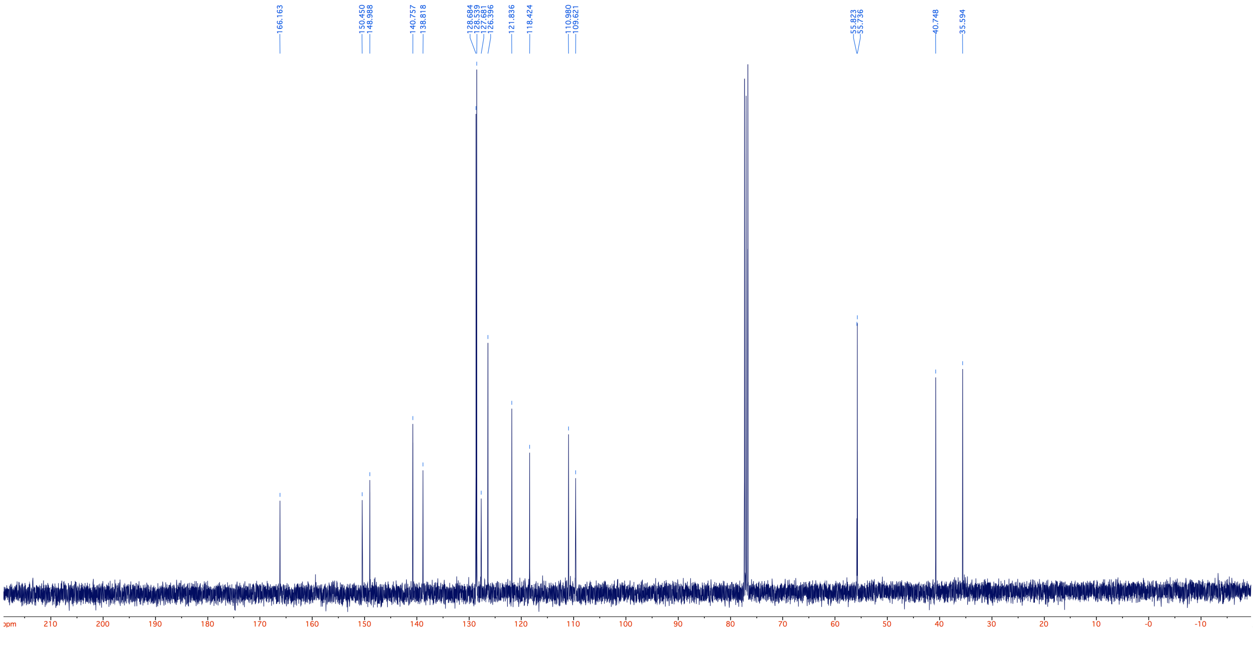

166.163
150.450
148.988
140.757
138.818
128.684
128.539
127.681
126.396
121.836
118.424
110.980
109.621
55.923
55.736
40.748
35.594
ppm
210
200
190
180
170
160
150
140
130
120
110
100
90
80
70
60
50
40
30
20
10
0
-10

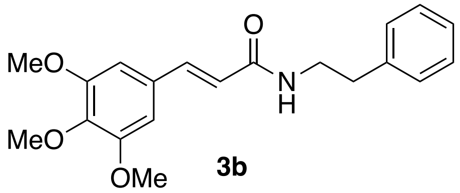

**3b**

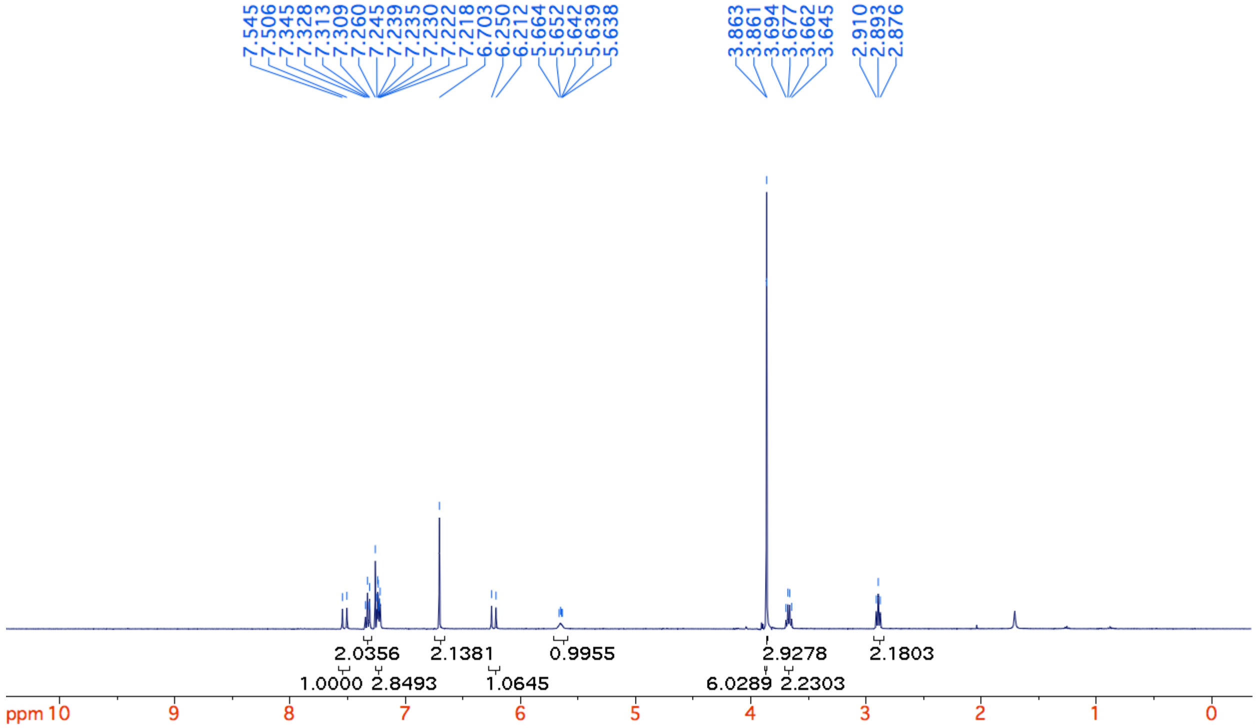
7.545
7.506
7.345
7.328
7.313
7.309
7.260
7.245
7.239
7.235
7.230
7.222
7.218
6.703
6.250
6.212
5.664
5.652
5.642
5.639
5.638
3.863
3.861
3.694
3.677
3.662
3.645
2.910
2.893
2.876
2.0356
1.0000
2.8493
2.1381
1.0645
0.9955
2.9278
6.0289
2.2303
2.1803
ppm 10
9
8
7
6
5
4
3
2
1
0

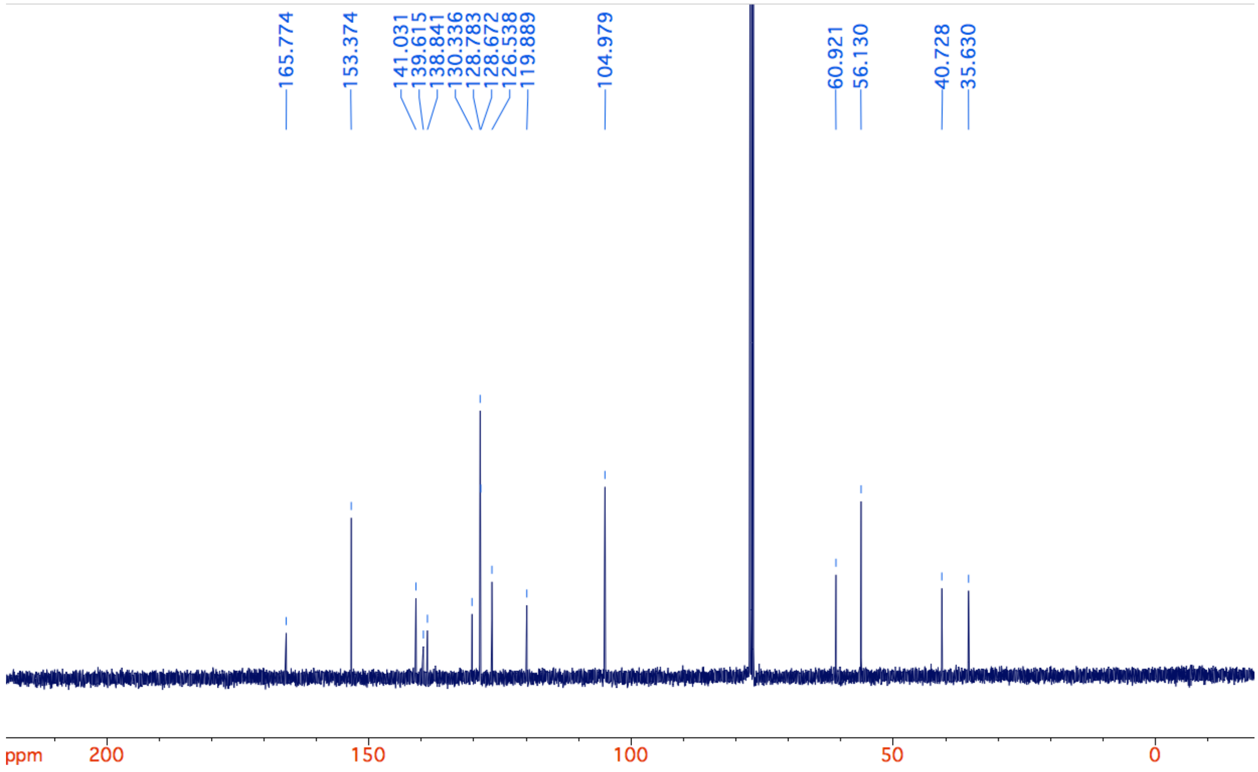
165.774
153.374
141.031
139.615
138.841
130.336
128.783
128.672
126.538
119.889
104.979
60.921
56.130
40.728
35.630
ppm
200
150
100
50
0

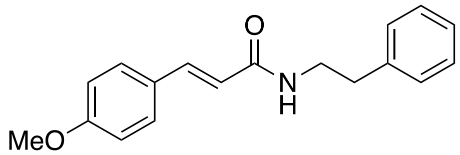

**3c**

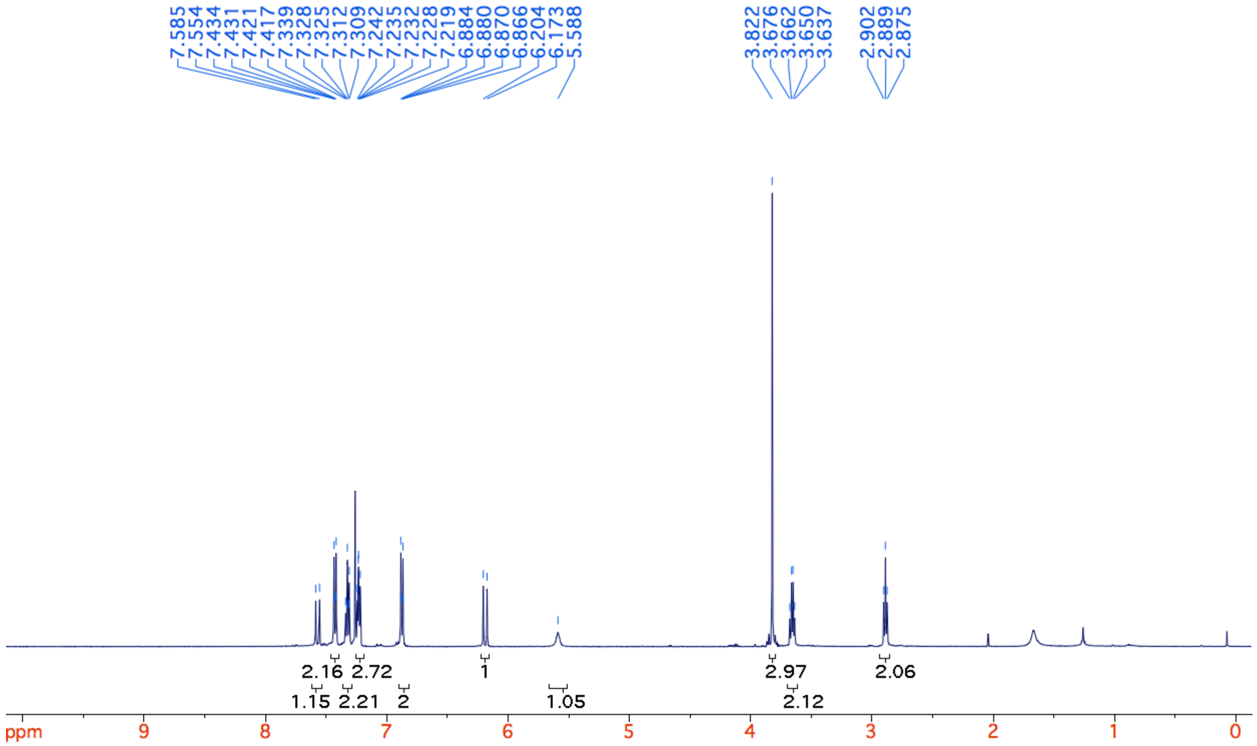
7.585
7.554
7.434
7.431
7.421
7.417
7.339
7.328
7.325
7.312
7.309
7.242
7.235
7.232
7.228
7.219
6.884
6.880
6.870
6.866
6.204
6.173
5.588
3.822
3.676
3.662
3.650
3.637
2.902
2.889
2.875
2.16
2.72
1
2.97
2.06
1.15
2.21
2
1.05
2.12
ppm

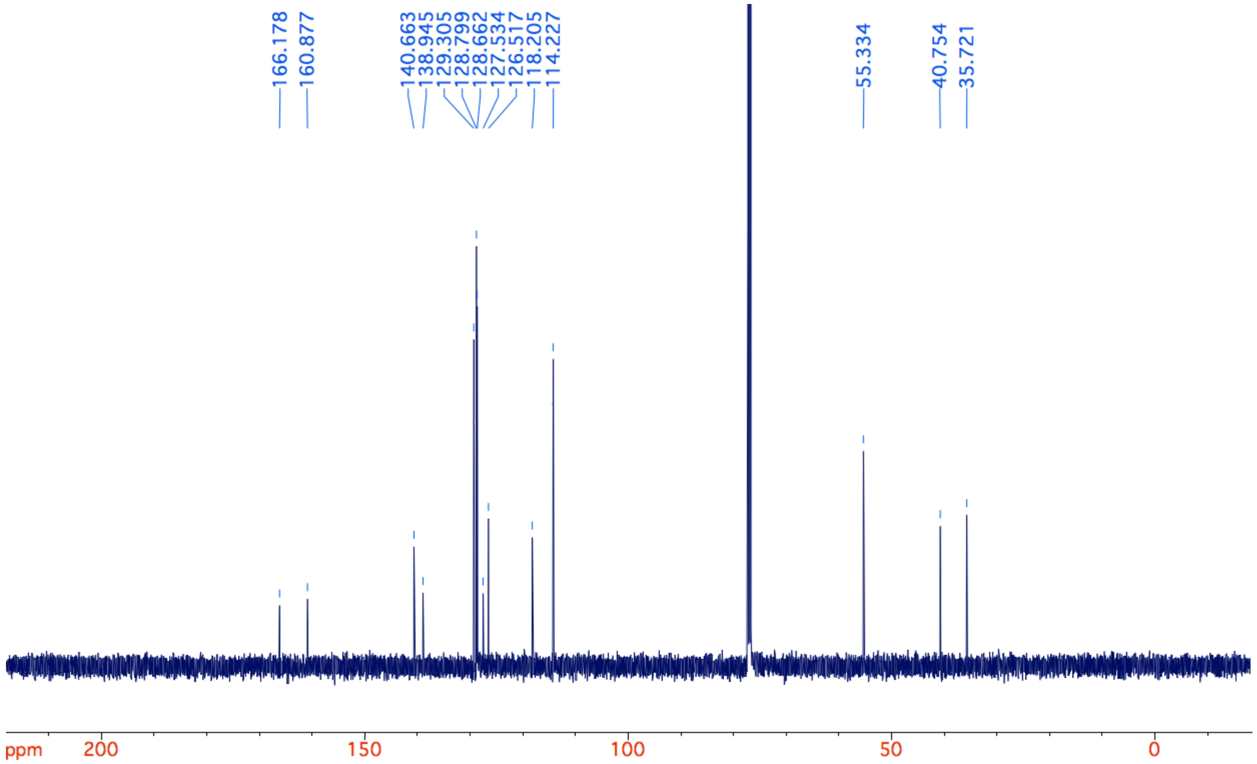
166.178
160.877
140.663
138.945
129.305
128.799
128.662
127.534
126.517
118.205
114.227
55.334
40.754
35.721
ppm

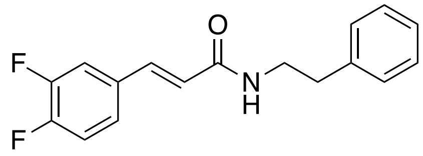

F
F
O
N
H


**3d**

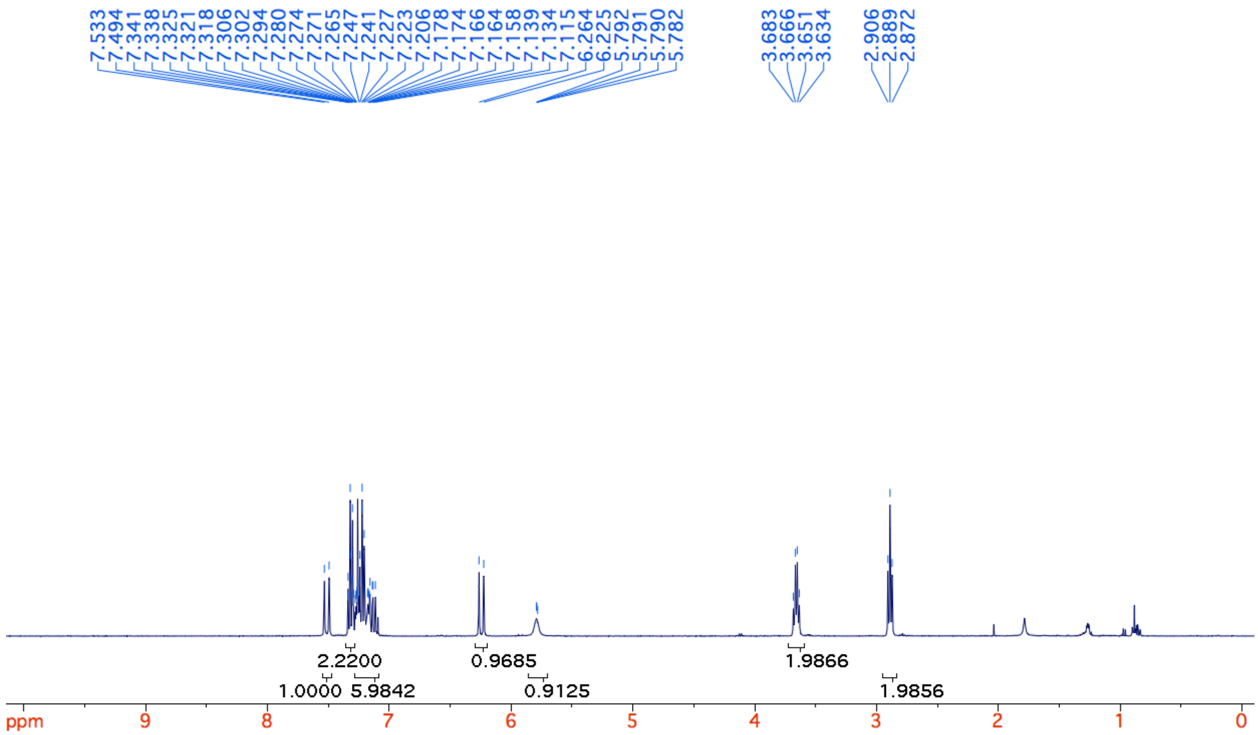

7.533
7.494
7.341
7.338
7.325
7.321
7.318
7.306
7.302
7.294
7.280
7.274
7.271
7.265
7.247
7.241
7.227
7.223
7.206
7.178
7.174
7.166
7.164
7.158
7.139
7.134
7.115
6.264
6.225
5.792
5.791
5.790
5.782
3.683
3.666
3.651
3.634
2.906
2.889
2.872
2.2200
0.9685
1.9866
1.0000
5.9842
0.9125
1.9856
ppm
9
8
7
6
5
4
3
2
1
0


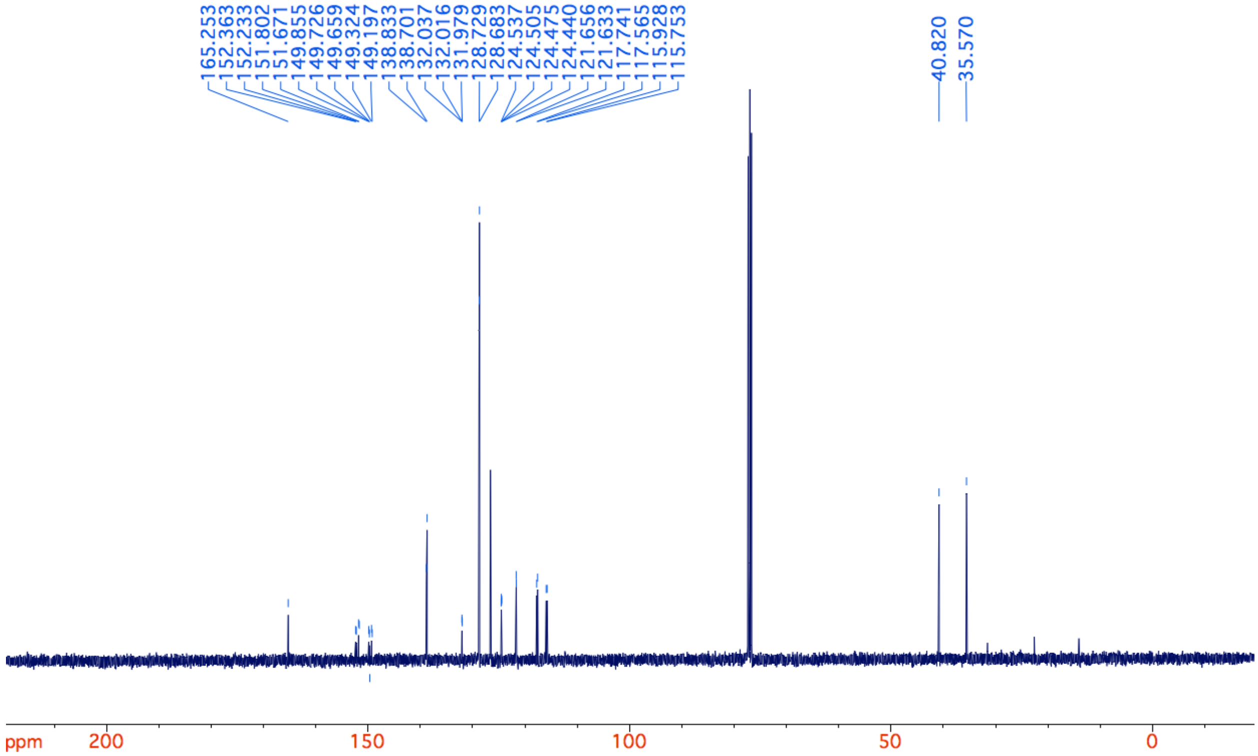

165.253
152.363
152.233
151.802
151.671
149.855
149.726
149.659
149.324
149.197
138.833
138.701
132.037
132.016
131.979
128.729
128.683
124.537
124.505
124.475
124.440
121.656
121.633
117.741
117.565
115.928
115.753
40.820
35.570
ppm
200
150
100
50
0

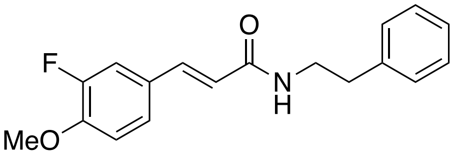


**3e**

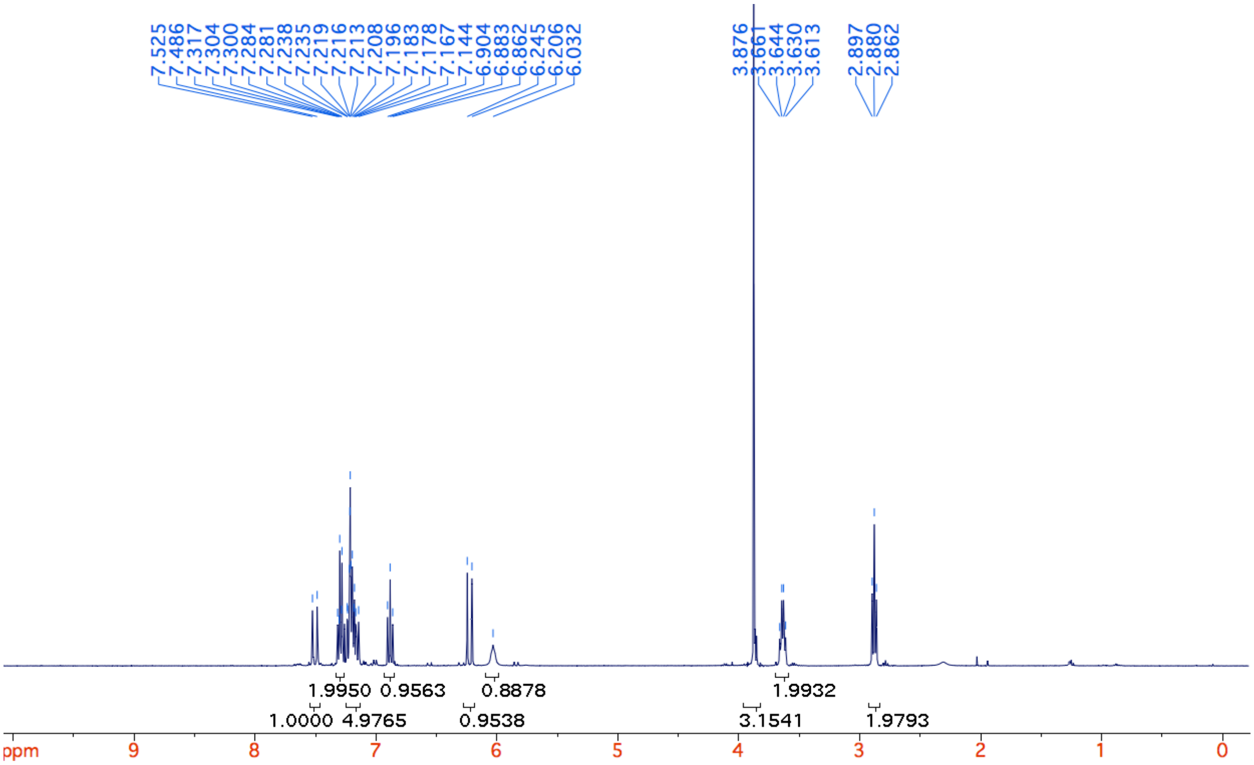

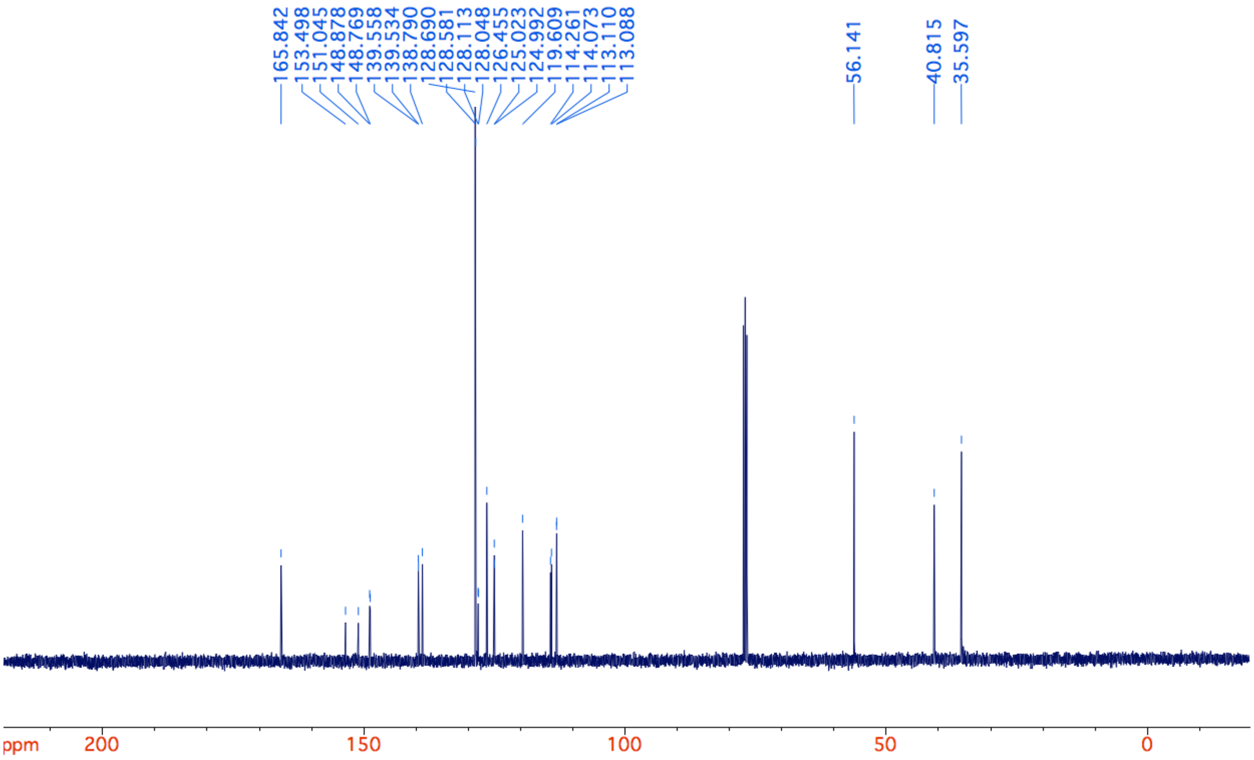

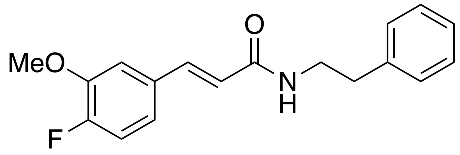


**3f**

(in $d_6$-acetone)

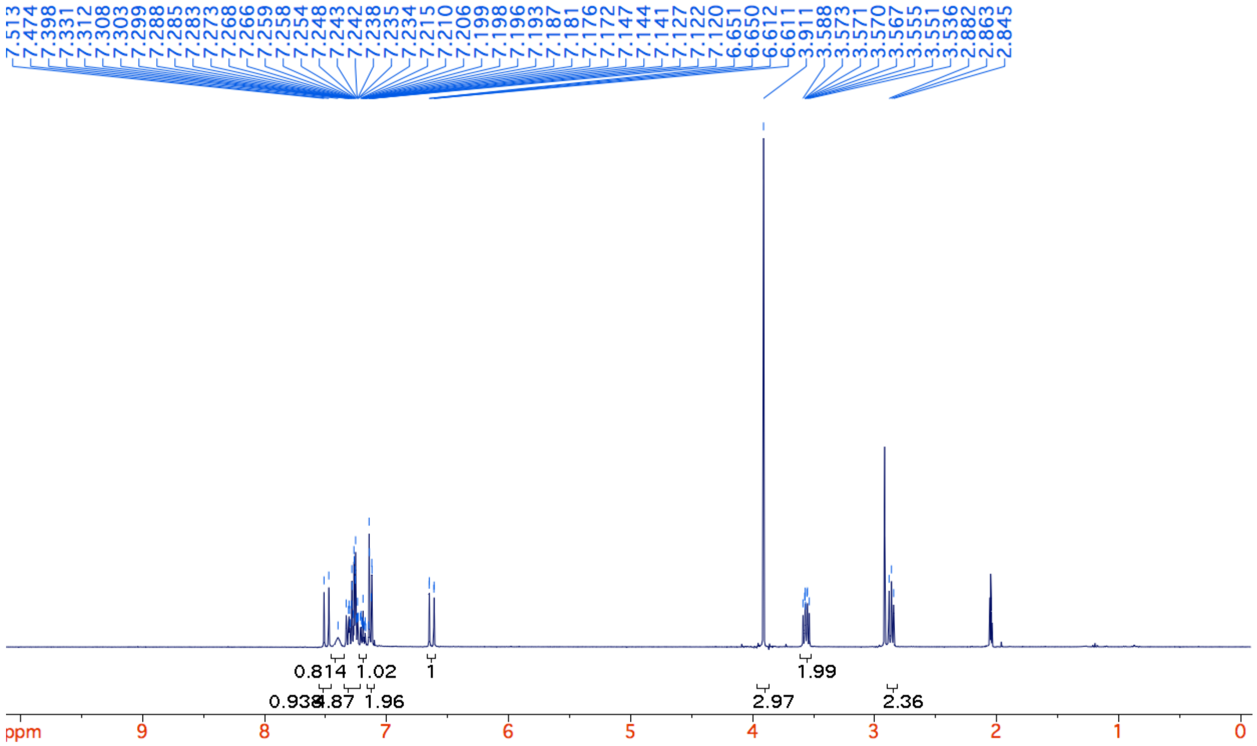


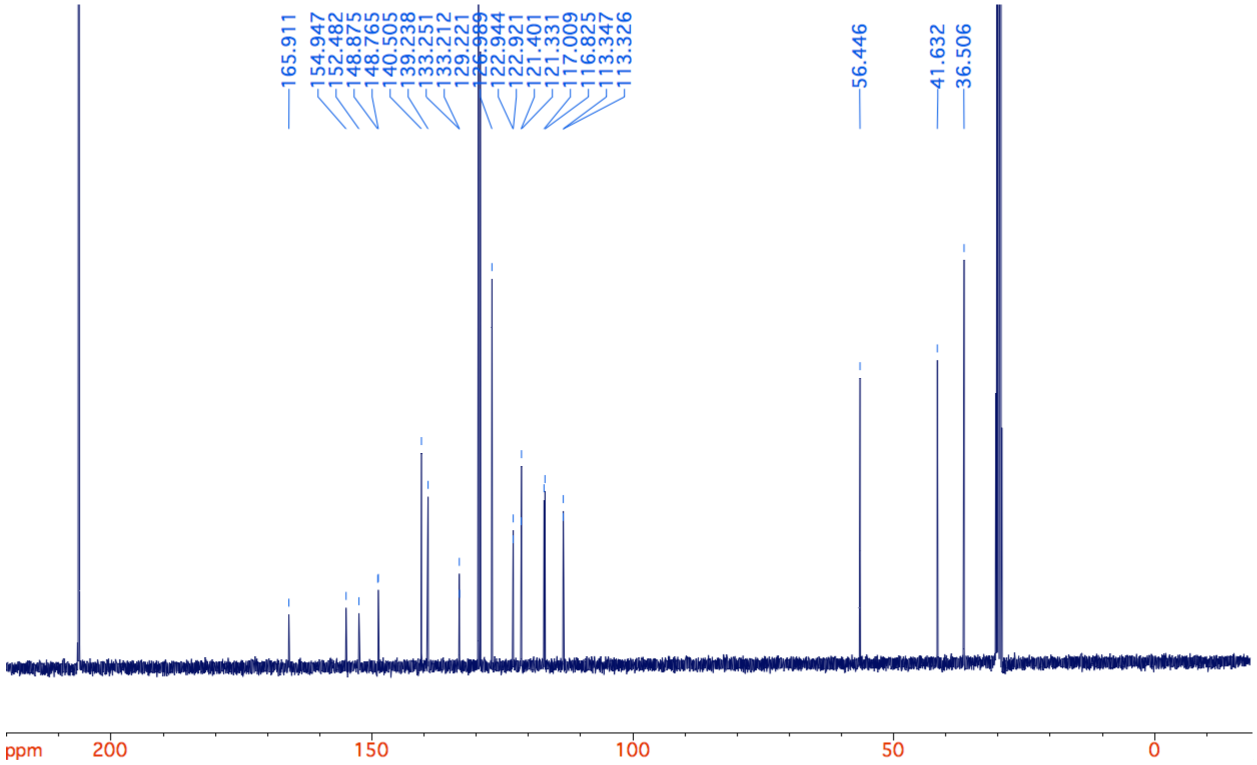

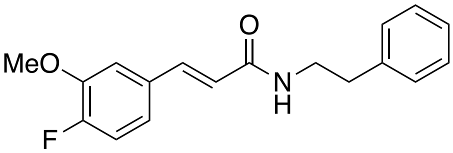
MeO
O
N
H
F

**3f**

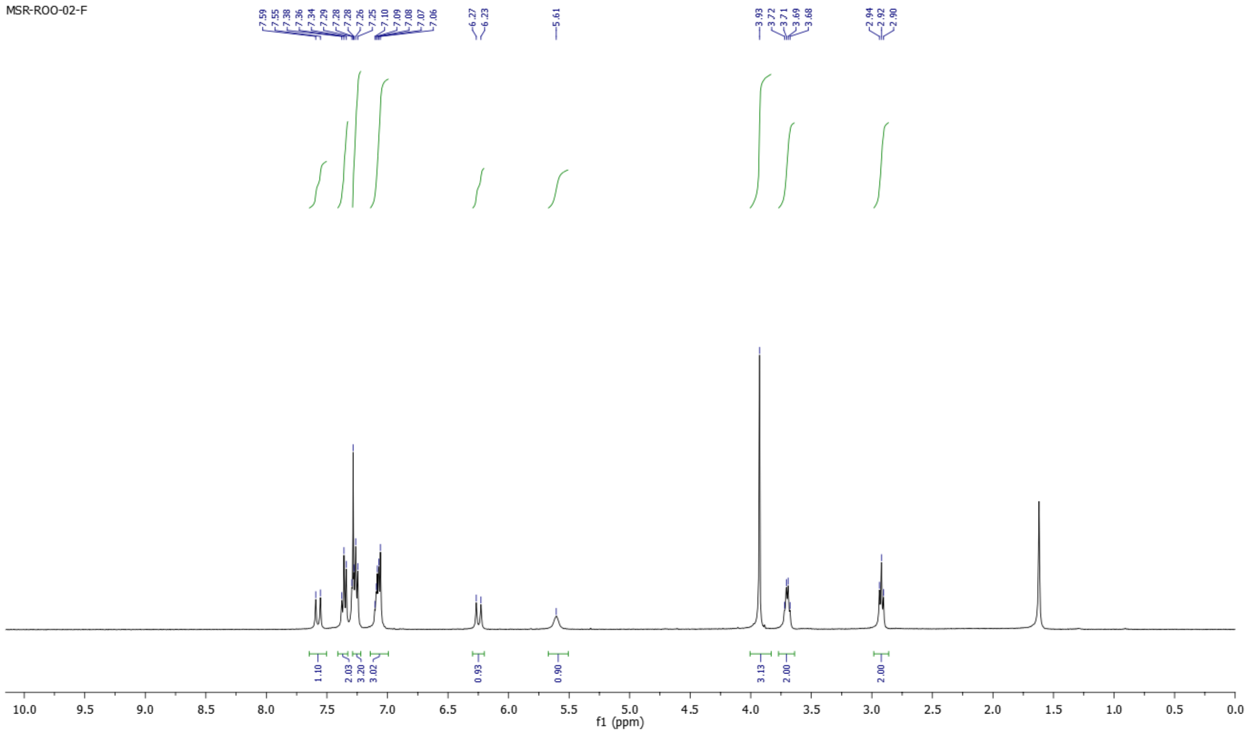
MSR-ROO-02-F
7.59 7.55 7.38 7.36 7.34 7.29 7.28 7.28 7.26 7.25 7.10 7.09 7.08 7.07 7.06
6.27 6.23
5.61
3.93 3.72 3.71 3.69 3.68
2.94 2.92 2.90
1.10 2.03 3.20 3.02 0.93 0.90 3.13 2.00 2.00
f1 (ppm)

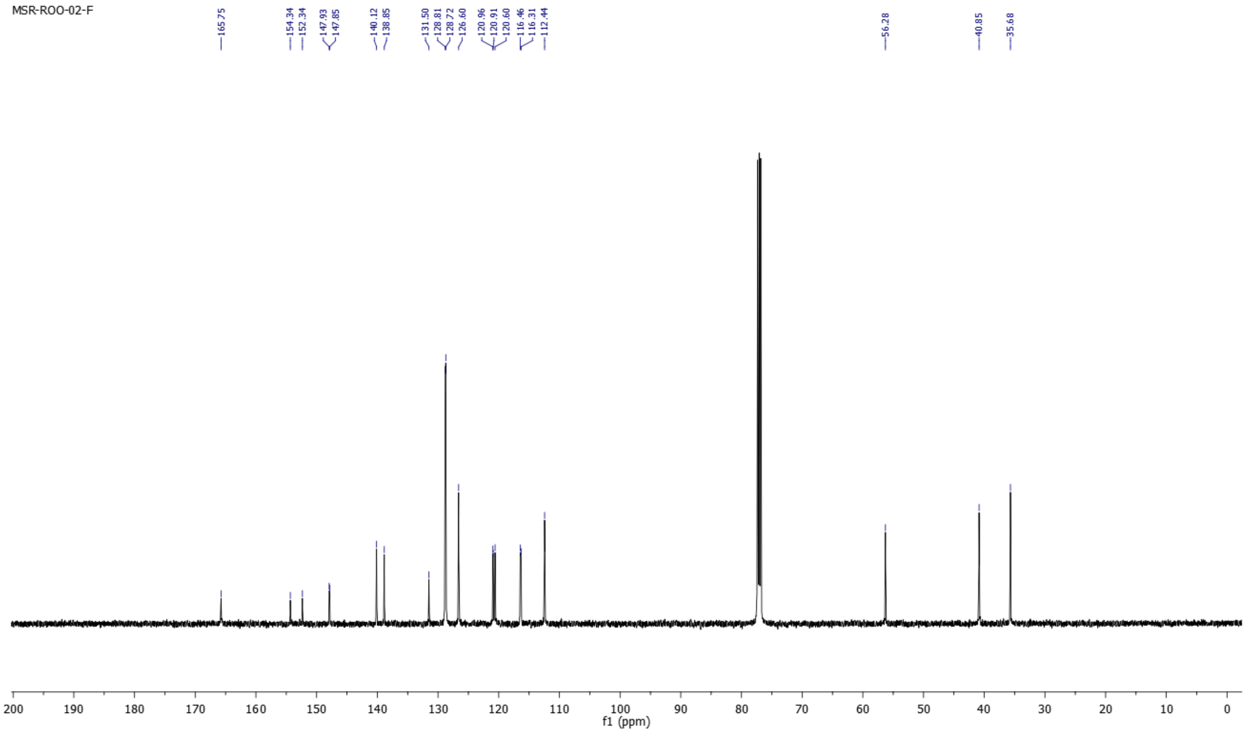
MSR-ROO-02-F
165.75
154.34 152.34 147.93 147.85
140.12 138.85
131.50 128.81 128.72 126.60
120.96 120.91 120.60 116.46 116.31 112.44
56.28
40.85 35.68
f1 (ppm)

MSR-AZB-02-19F-1

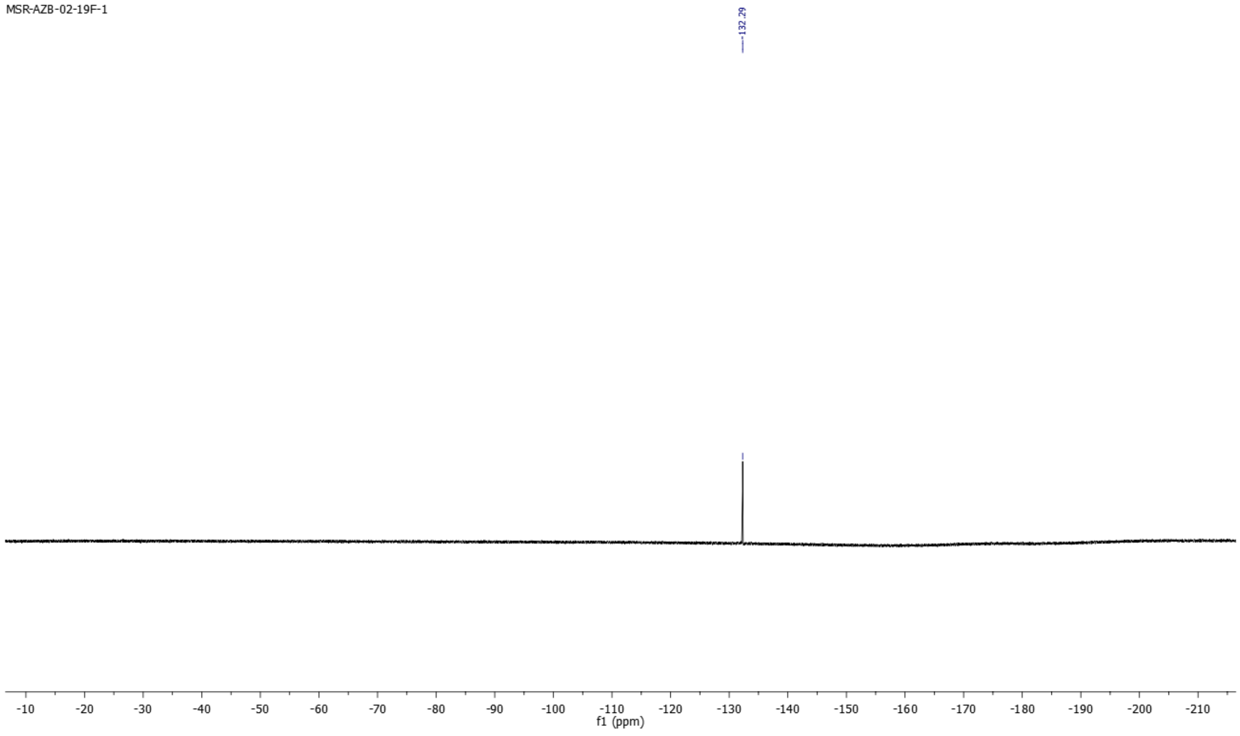

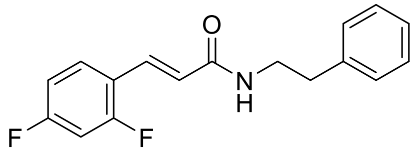

**3g**

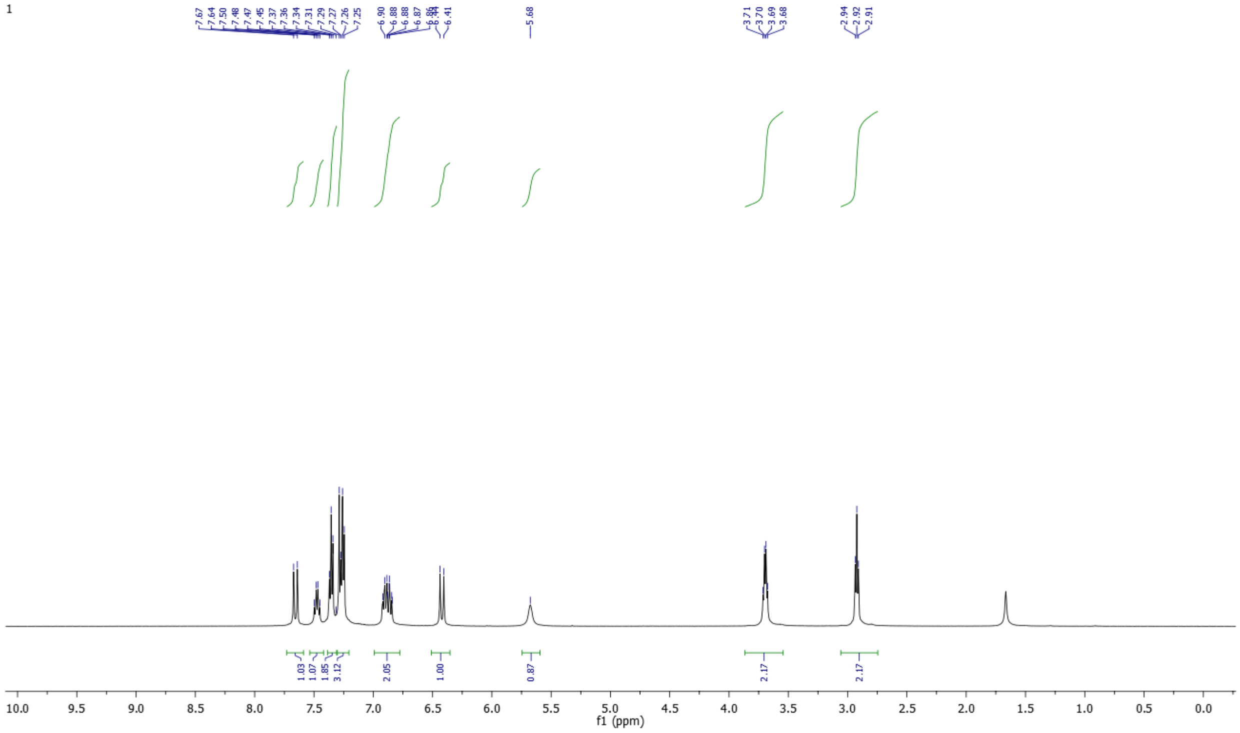

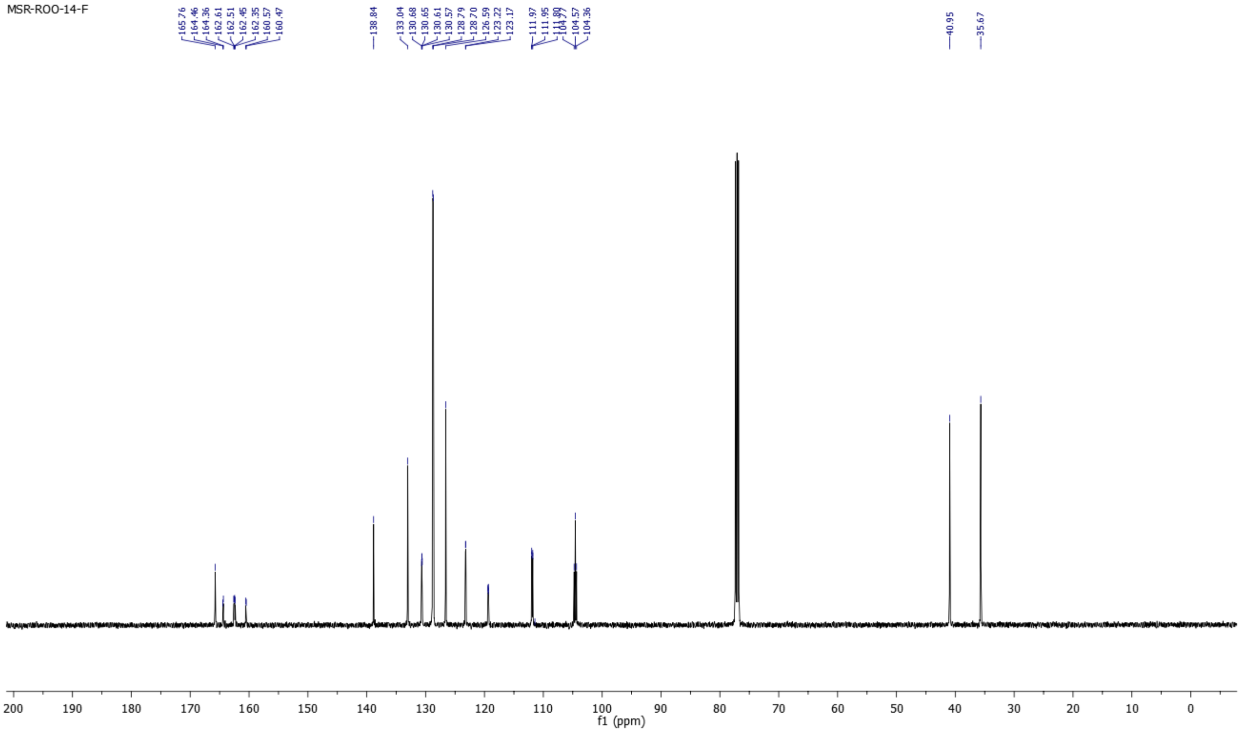


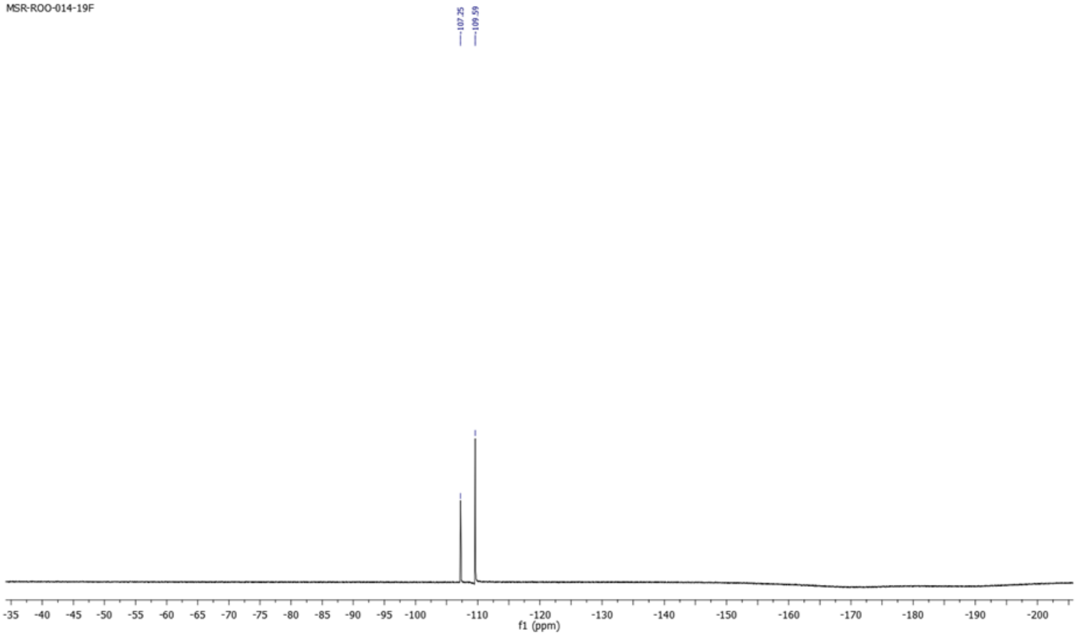


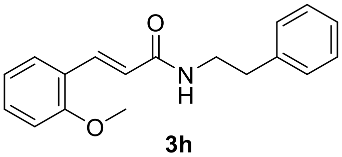

**3h**

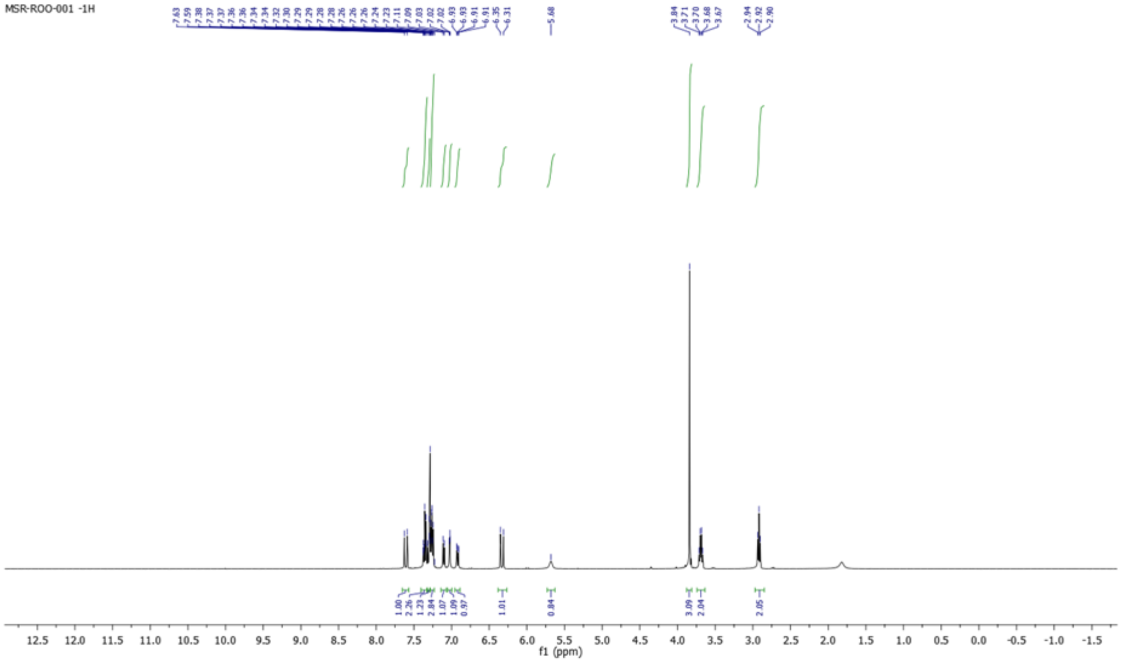


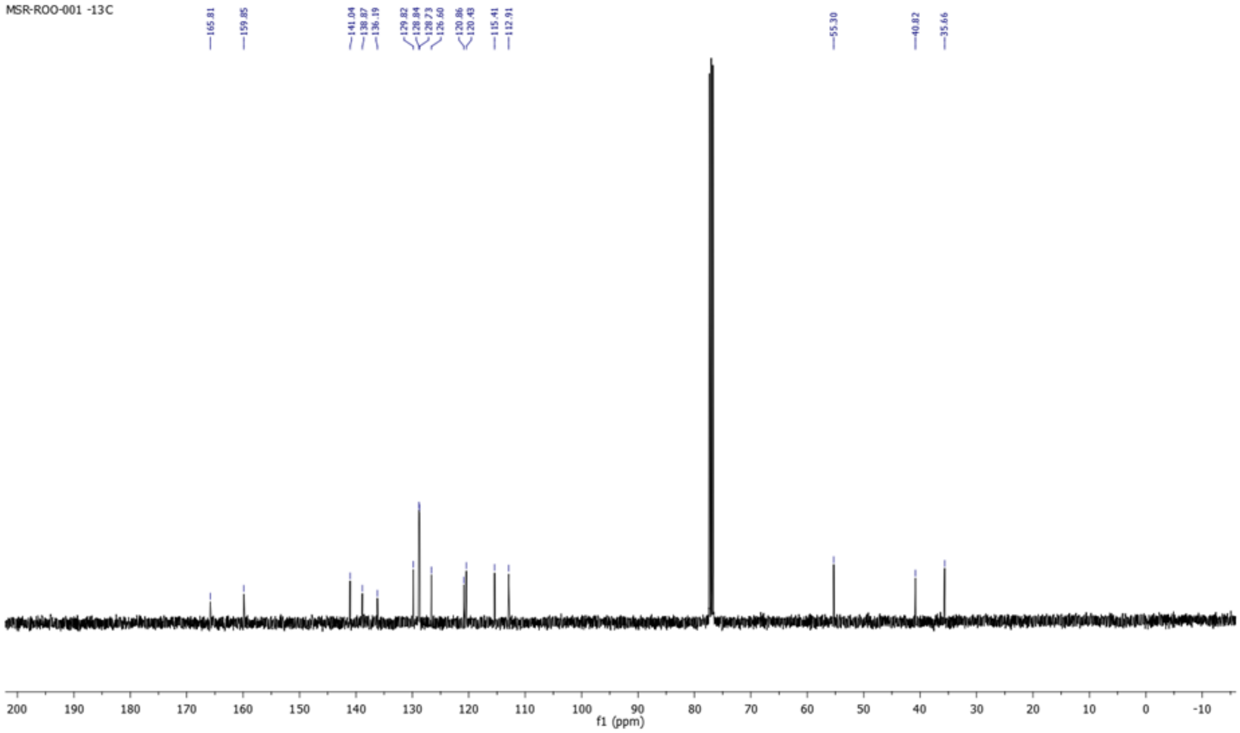


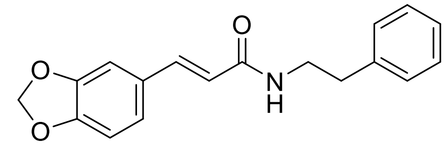

**3i**

MJ-CS-005 1H

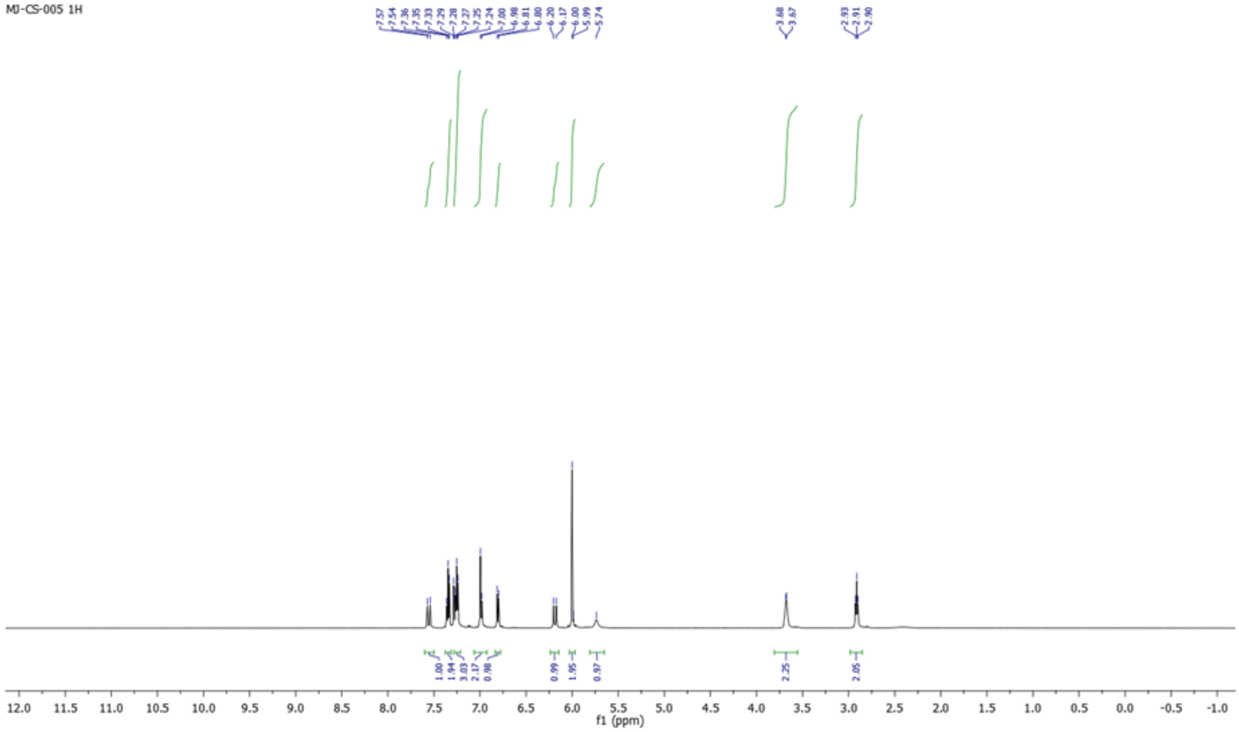

MJ-CS-005 13C

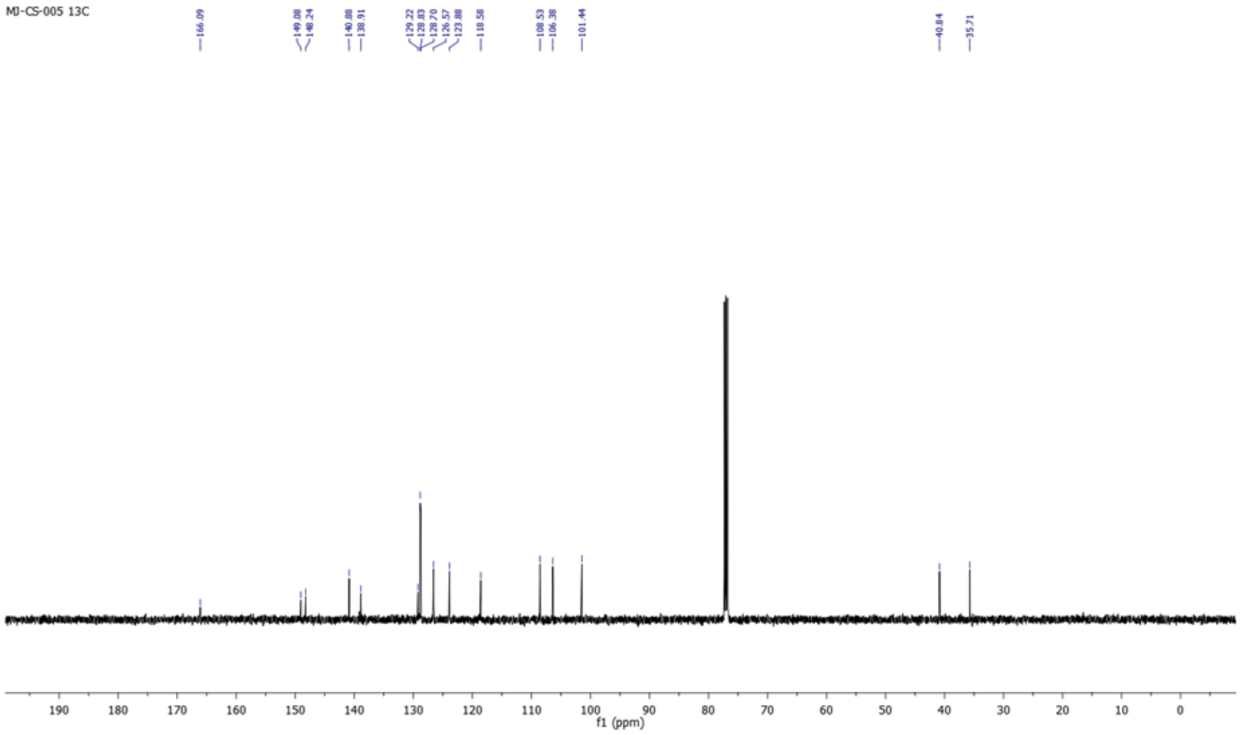

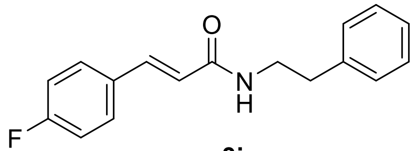

**3j**

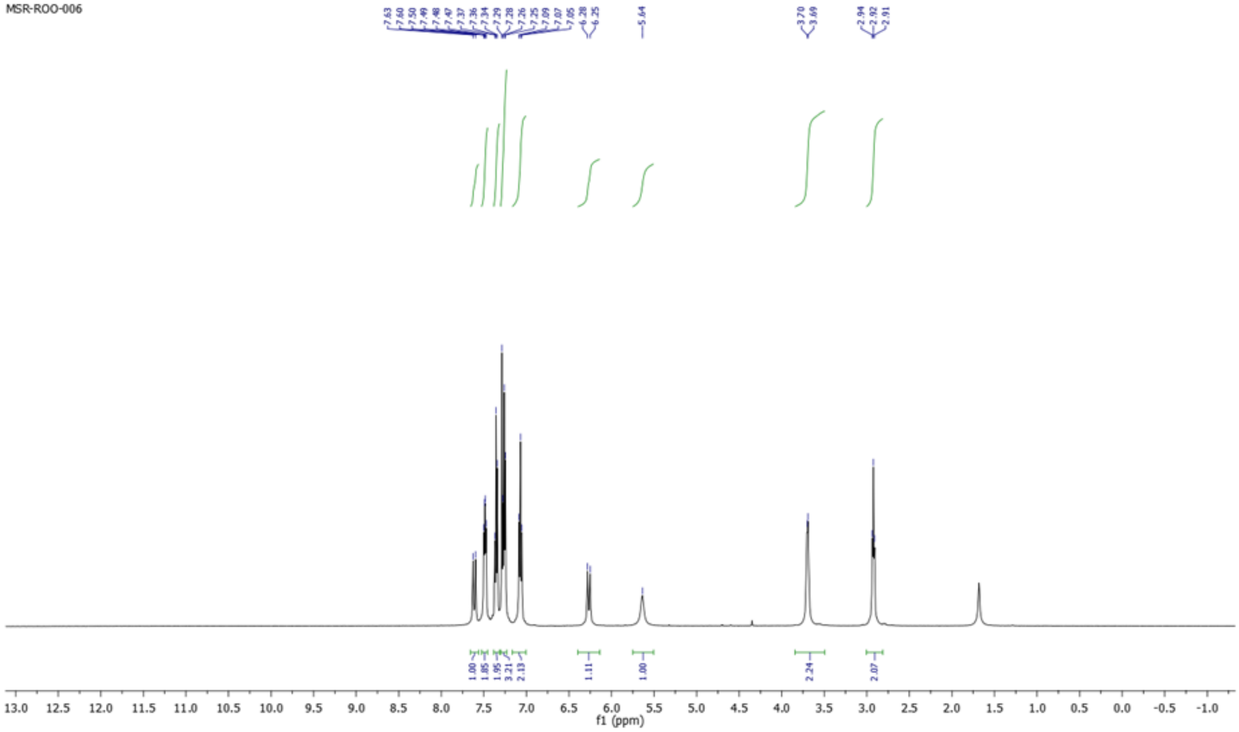
MSR-ROO-006
7.63 7.60 7.50 7.49 7.48 7.47 7.37 7.36 7.34 7.29 7.28 7.26 7.25 7.09 7.07 7.05 6.28 6.25
5.64
3.70 3.69
2.94 2.92 2.91
1.00 1.85 1.95 3.21 2.13 1.11 1.00 2.24 2.07
f1 (ppm)

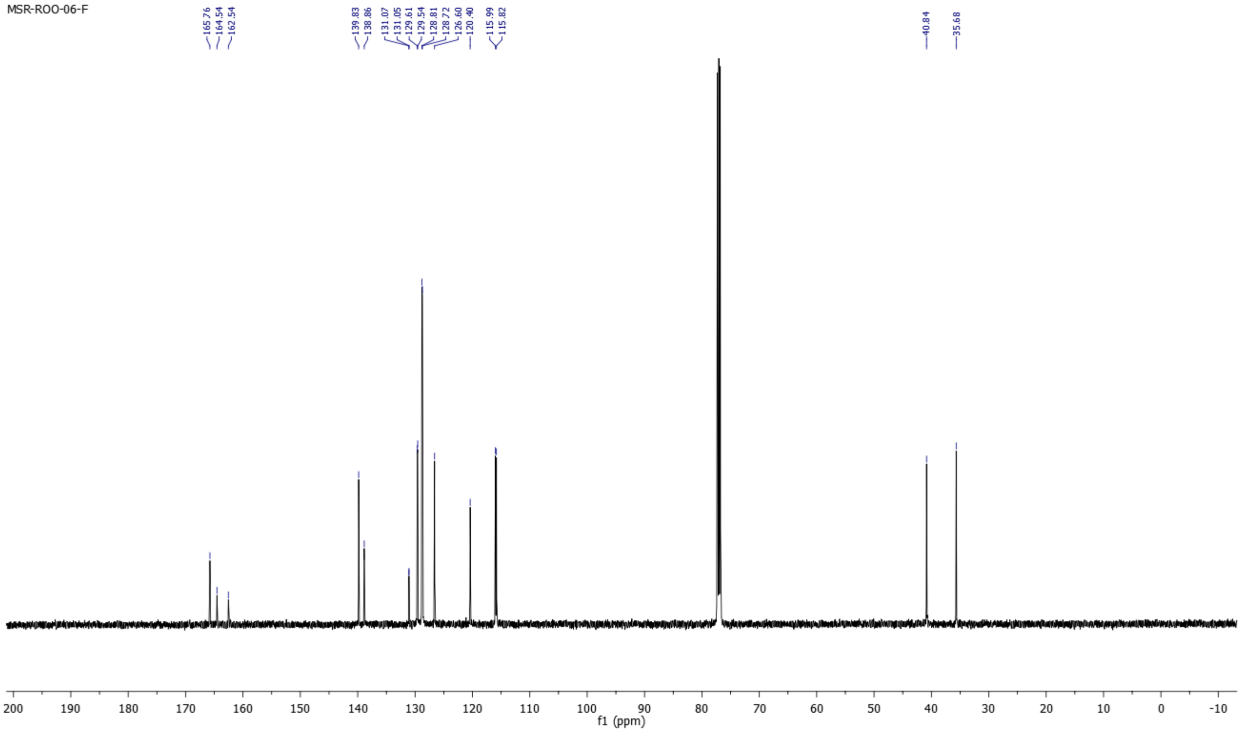
MSR-ROO-06-F
165.76 164.54 162.54
139.83 138.86 131.07 131.05 129.61 129.54 128.81 128.72 126.60 120.40 115.99 115.82
40.84
35.68
f1 (ppm)

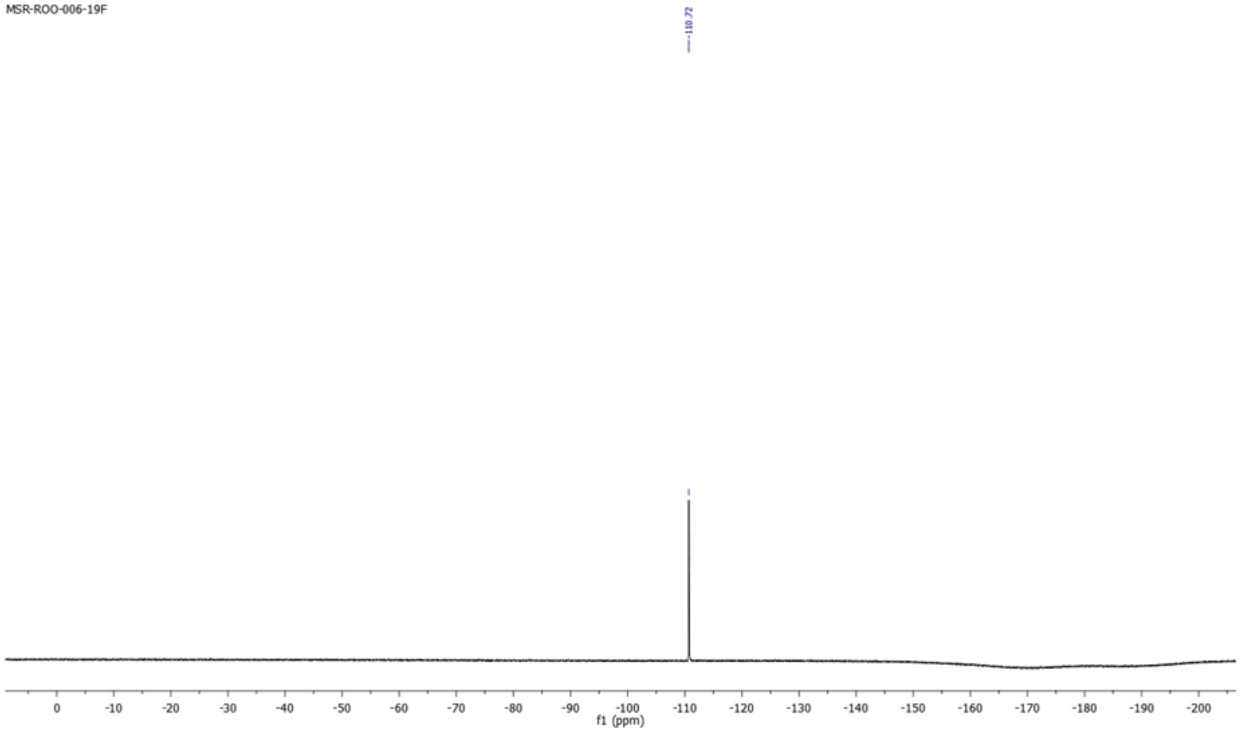

MSR-ROO-006-19F
-110.72
0
-10
-20
-30
-40
-50
-60
-70
-80
-90
-100
-110
-120
-130
-140
-150
-160
-170
-180
-190
-200
f1 (ppm)

## Cell Viability Curves of CAPE, CAPA, and Analogues against HeLa Cells

**CAPE**

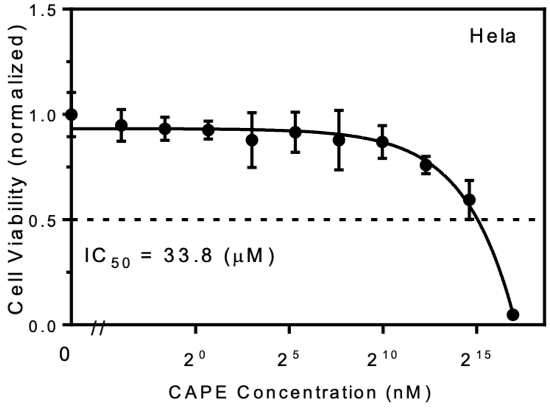


**CAPA**

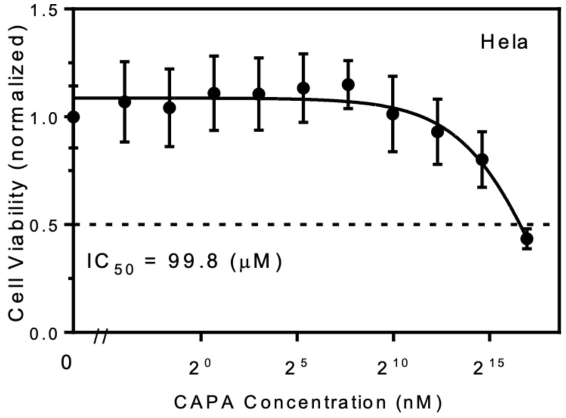


**3a**

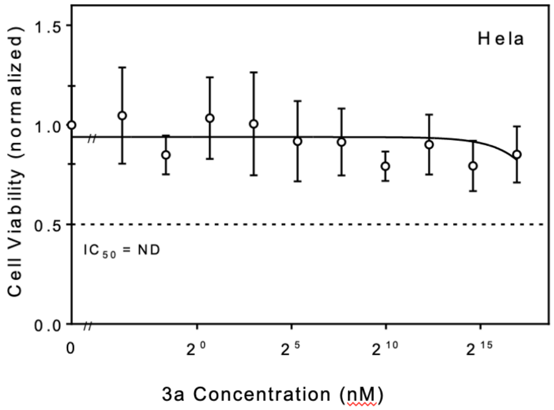

**3b**

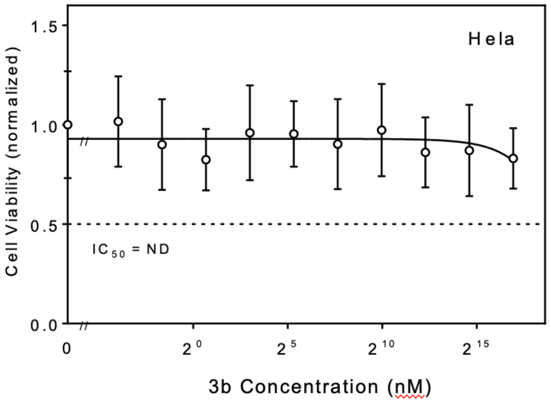
Hela
Cell Viability (normalized)
1.5
1.0
0.5
0.0
IC50 = ND
0
2 0
2 5
2 10
2 15
3b Concentration (nM)

**3c**

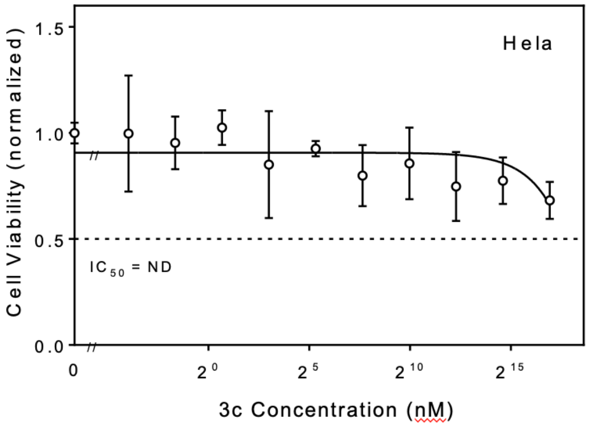
Hela
Cell Viability (normalized)
1.5
1.0
0.5
0.0
IC50 = ND
0
2 0
2 5
2 10
2 15
3c Concentration (nM)

**3d**

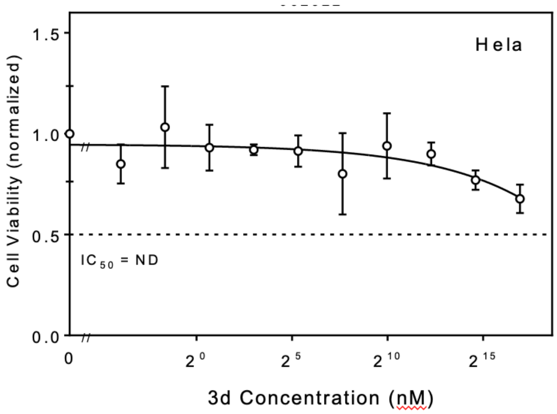
Hela
Cell Viability (normalized)
1.5
1.0
0.5
0.0
IC50 = ND
0
2 0
2 5
2 10
2 15
3d Concentration (nM)

**3e**

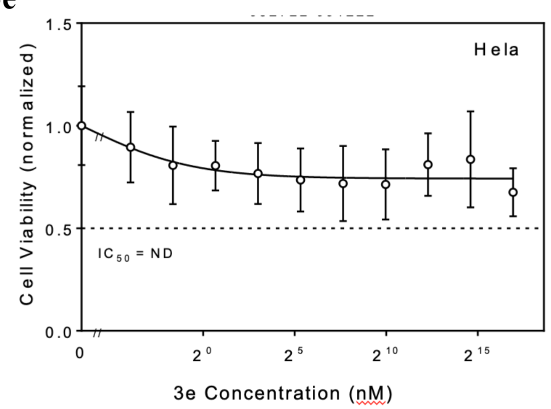
Hela
Cell Viability (normalized)
1.5
1.0
0.5
0.0
IC 50 = ND
0
2 0
2 5
2 10
2 15
3e Concentration (nM)

**3f**

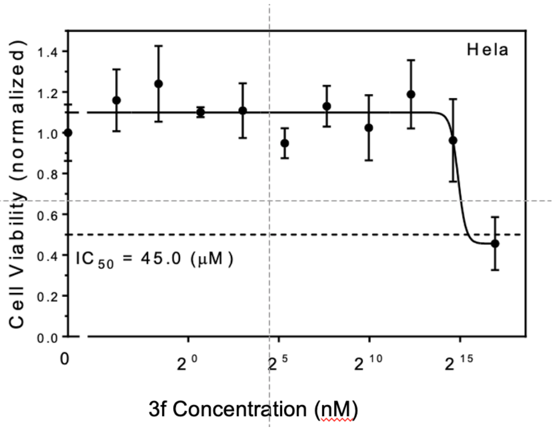
Hela
Cell Viability (normalized)
1.4
1.2
1.0
0.8
0.6
0.4
0.2
0.0
IC 50 = 45.0 (μM)
0
2 0
2 5
2 10
2 15
3f Concentration (nM)

**3g**

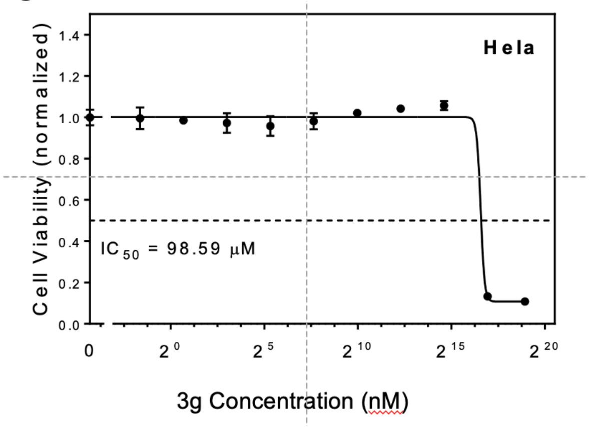
Hela
Cell Viability (normalized)
1.4
1.2
1.0
0.8
0.6
0.4
0.2
0.0
IC 50 = 98.59 μM
0
2 0
2 5
2 10
2 15
2 20
3g Concentration (nM)

**3h**

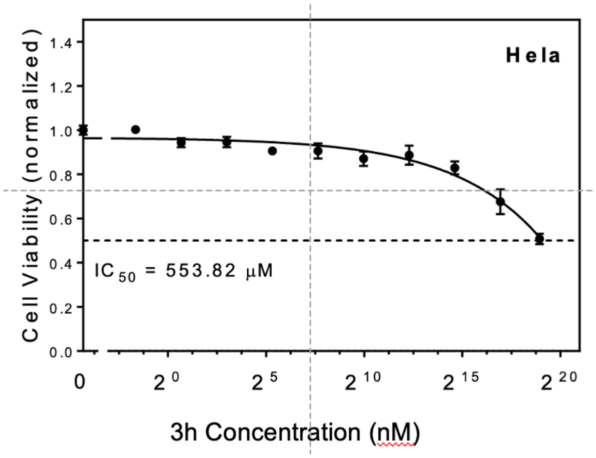
Hela
IC 50 = 553.82 µM
Cell Viability (normalized)
1.4
1.2
1.0
0.8
0.6
0.4
0.2
0.0
0
2 0
2 5
2 10
2 15
2 20
3h Concentration (nM)

**3i**

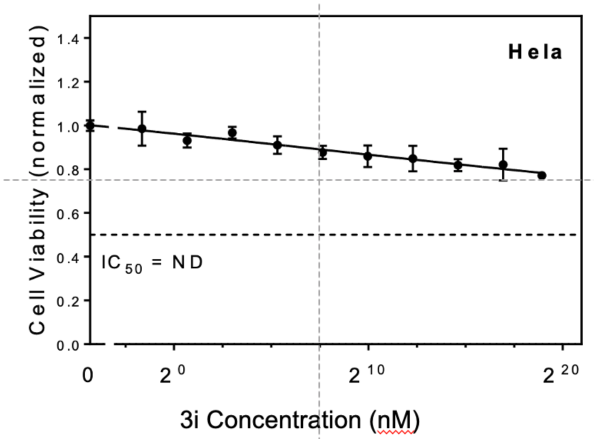
Hela
IC 50 = ND
Cell Viability (normalized)
1.4
1.2
1.0
0.8
0.6
0.4
0.2
0.0
0
2 0
2 10
2 20
3i Concentration (nM)

**3j**

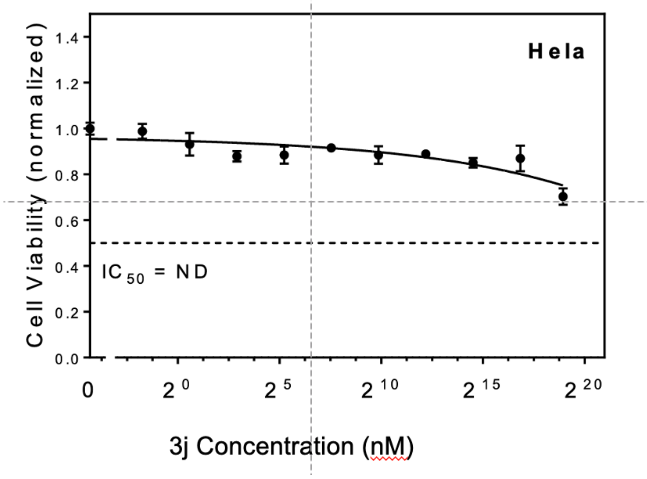
Hela
IC 50 = ND
Cell Viability (normalized)
1.4
1.2
1.0
0.8
0.6
0.4
0.2
0.0
0
2 0
2 5
2 10
2 15
2 20
3j Concentration (nM)

## Cell Viability Curves of CAPE, CAPA, and Analogues against BE(2)-C Cells

**CAPE**

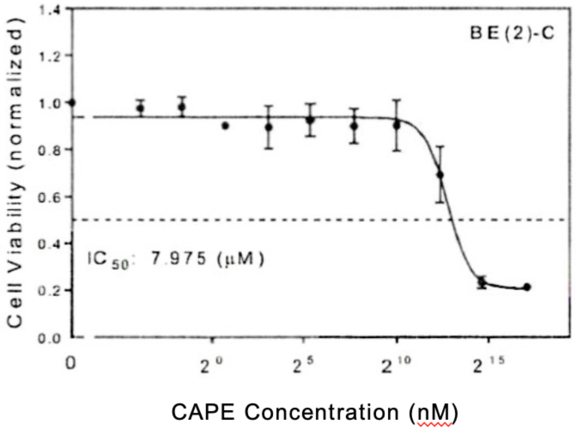


**CAPA**

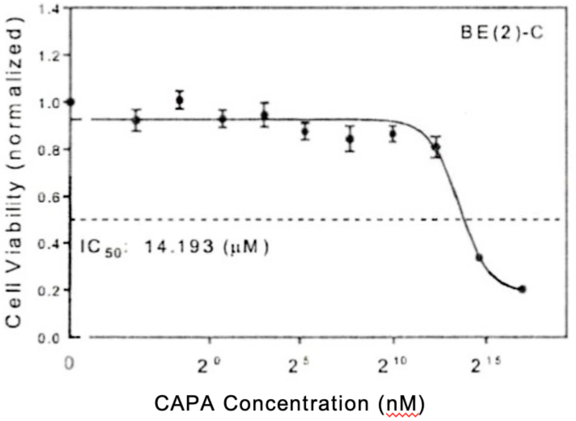

**3a**

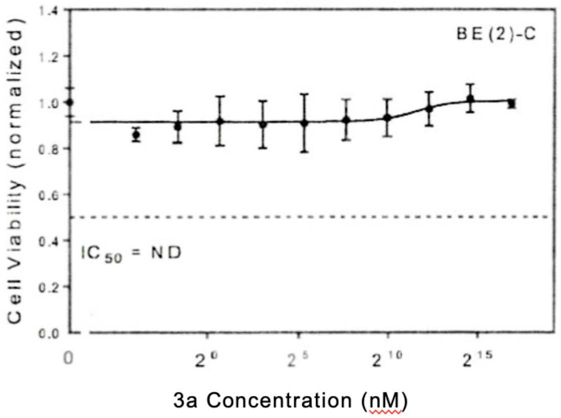
BE(2)-C
Cell Viability (normalized)
1.4
1.2
1.0
0.8
0.6
0.4
0.2
0.0
$IC_{50}$ = ND
0
$2^0$
$2^5$
$2^{10}$
$2^{15}$
3a Concentration (nM)

**3d**

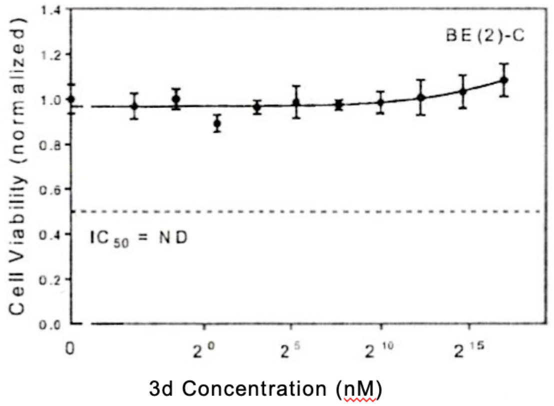
BE(2)-C
Cell Viability (normalized)
1.4
1.2
1.0
0.8
0.6
0.4
0.2
0.0
$IC_{50}$ = ND
0
$2^0$
$2^5$
$2^{10}$
$2^{15}$
3d Concentration (nM)

**3e**

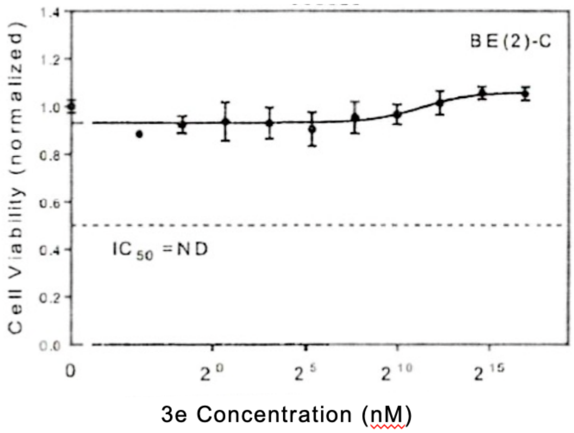
BE(2)-C
Cell Viability (normalized)
1.4
1.2
1.0
0.8
0.6
0.4
0.2
0.0
$IC_{50}$ =ND
0
$2^0$
$2^5$
$2^{10}$
$2^{15}$
3e Concentration (nM)

**3f**

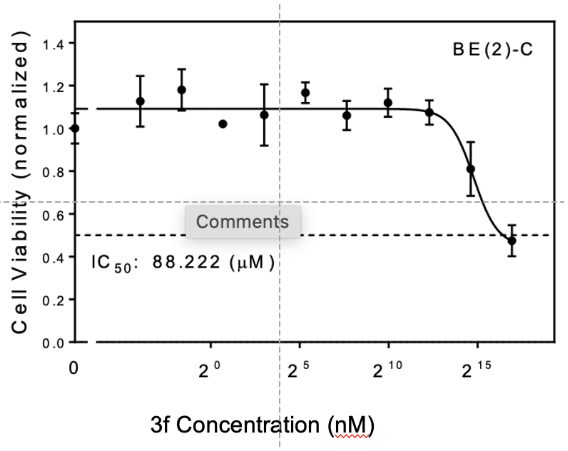
BE(2)-C
Cell Viability (normalized)
1.4
1.2
1.0
0.8
0.6
0.4
0.2
0.0
IC 50: 88.222 (μM)
0
2 0
2 5
2 10
2 15
3f Concentration (nM)

**3g**

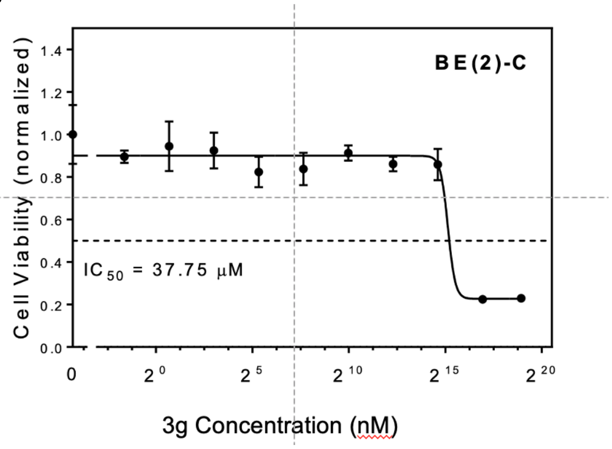
BE(2)-C
Cell Viability (normalized)
1.4
1.2
1.0
0.8
0.6
0.4
0.2
0.0
IC 50 = 37.75 μM
0
2 0
2 5
2 10
2 15
2 20
3g Concentration (nM)

**3h**

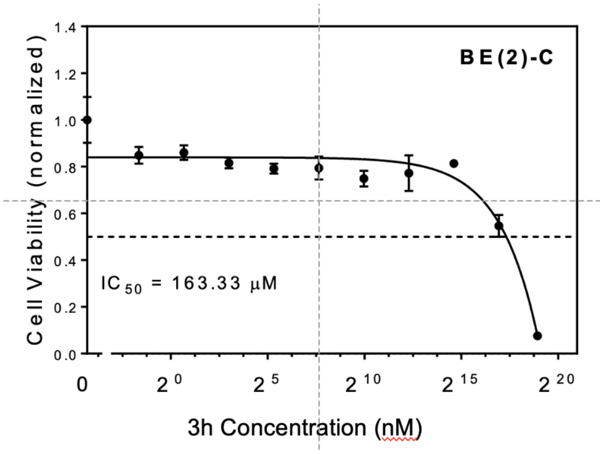
BE(2)-C
Cell Viability (normalized)
1.4
1.2
1.0
0.8
0.6
0.4
0.2
0.0
IC 50 = 163.33 μM
0
2 0
2 5
2 10
2 15
2 20
3h Concentration (nM)

**3i**

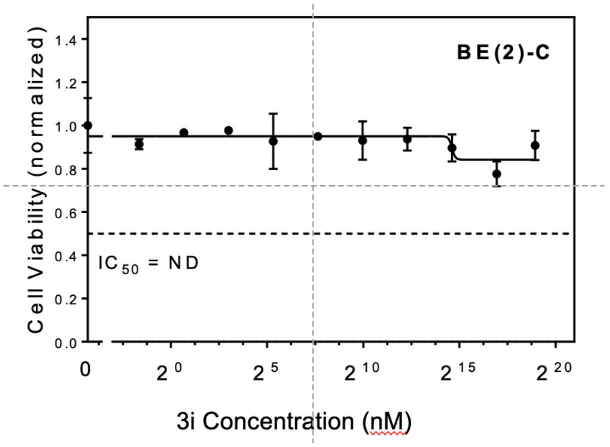
BE(2)-C
Cell Viability (normalized)
1.4
1.2
1.0
0.8
0.6
0.4
0.2
0.0
IC 50 = ND
0
2 0
2 5
2 10
2 15
2 20
3i Concentration (nM)

**3j**

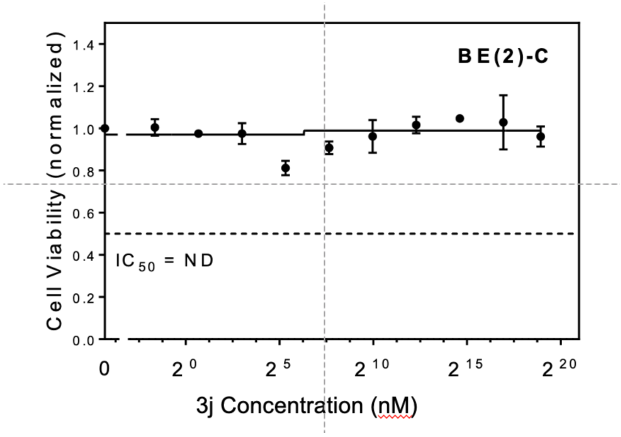
BE(2)-C
Cell Viability (normalized)
1.4
1.2
1.0
0.8
0.6
0.4
0.2
0.0
IC 50 = ND
0
2 0
2 5
2 10
2 15
2 20
3j Concentration (nM)

## Statistical Analysis of $IC_{50}$ Data

| | BE(2)-C | | Hela | | ***p* value** |
|---|---|---|---|---|---|
| | Mean | SD | Mean | SD | |
| **CAPE** | 5.186 | 2.173 | 32.34 | 12.336 | **0.02** |
| **CAPA** | 12.152 | 2.887 | 111.957 | 17.192 | **0.015** |
| **3f** | 90.954 | 52.653 | 63.509 | 29.385 | **0.470** |
| **3g** | 91.842 | 48.338 | 140.671 | 58.096 | **0.380** |
| **3h** | 162.877 | 0.641 | 600.276 | 65.699 | **0.01** |

Table S1. Two-tailed t-test *p* values for $IC_{50}$ value comparisons for BE(2)-C versus HeLa cells for CAPE, CAPA, and CAPA analogues **3f**, **3g**, and **3h.**

CAPA

E = -938.503411770 H

Calculated Lowest Freq = 15.385 $cm^{-1}$

ZPE = 0.307751 H

Sum of electronic and thermal Enthalpies = -938.175120 H

| | | | | |
|---|---|---|---|---|
| 1 | 6 | 0.000001306 | -0.000000300 | -0.000000607 |
| 2 | 6 | -0.000000348 | -0.000000867 | -0.000001210 |
| 3 | 6 | -0.000000343 | 0.000001298 | 0.000000507 |
| 4 | 6 | 0.000000250 | -0.000000643 | -0.000000382 |
| 5 | 6 | 0.000000627 | 0.000000387 | 0.000000339 |
| 6 | 6 | 0.000000204 | -0.000001294 | -0.000001202 |
| 7 | 1 | 0.000000294 | 0.000000251 | 0.000000100 |
| 8 | 1 | -0.000000038 | 0.000000745 | -0.000000416 |
| 9 | 1 | -0.000000198 | -0.000000029 | -0.000000026 |
| 10 | 6 | -0.000000137 | -0.000001768 | 0.000001200 |
| 11 | 6 | 0.000000517 | 0.000000221 | 0.000001005 |
| 12 | 1 | 0.000000036 | 0.000000678 | -0.000000346 |
| 13 | 1 | 0.000000872 | 0.000000513 | -0.000000071 |
| 14 | 6 | 0.000000750 | 0.000002365 | -0.000000888 |
| 15 | 8 | -0.000002149 | 0.000000263 | -0.000000968 |
| 16 | 7 | -0.000003281 | -0.000006469 | 0.000000454 |
| 17 | 1 | 0.000003272 | 0.000002005 | 0.000000856 |
| 18 | 6 | -0.000000488 | 0.000004543 | -0.000002029 |
| 19 | 1 | -0.000000956 | 0.000000700 | -0.000001305 |
| 20 | 1 | 0.000000220 | 0.000000092 | -0.000000100 |
| 21 | 6 | 0.000001221 | -0.000007859 | 0.000004671 |
| 22 | 1 | -0.000000326 | 0.000001995 | -0.000000553 |
| 23 | 1 | -0.000000716 | 0.000000799 | 0.000000395 |
| 24 | 6 | 0.000001081 | -0.000001200 | -0.000006036 |
| 25 | 6 | 0.000001730 | 0.000000980 | 0.000002350 |
| 26 | 6 | -0.000003226 | 0.000001586 | 0.000002076 |
| 27 | 6 | -0.000002198 | 0.000000672 | 0.000001785 |
| 28 | 1 | 0.000000025 | -0.000000361 | -0.000000143 |
| 29 | 6 | 0.000003945 | 0.000001803 | 0.000002016 |
| 30 | 1 | 0.000000433 | -0.000000258 | 0.000000135 |
| 31 | 6 | -0.000000621 | -0.000002052 | -0.000003070 |
| 32 | 1 | 0.000000220 | -0.000000316 | -0.000000138 |
| 33 | 1 | -0.000000198 | -0.000000161 | -0.000000193 |
| 34 | 1 | -0.000000004 | 0.000000137 | 0.000000125 |
| 35 | 8 | -0.000000598 | 0.000001009 | 0.000000892 |
| 36 | 1 | -0.000000601 | -0.000000438 | -0.000000406 |
| 37 | 8 | -0.000000075 | 0.000000599 | 0.000000622 |
| 38 | 1 | -0.000000501 | 0.000000368 | 0.000000559 |

H2-CAPA

E = -939.673319 H

Calculated Lowest Freq = 13.0234 $cm^{-1}$

ZPE = 0.330472 H

Sum of electronic and thermal Enthalpies = -939.321789 H

| | | | | | |
|---|---|---|---|---|---|
| 1 | 6 | 0 | -5.334471 | -0.180103 | 0.091368 |
| 2 | 6 | 0 | -4.272162 | 0.711767 | 0.050146 |
| 3 | 6 | 0 | -2.948120 | 0.259329 | -0.113472 |
| 4 | 6 | 0 | -2.740014 | -1.126236 | -0.225299 |
| 5 | 6 | 0 | -3.804242 | -2.022975 | -0.183465 |
| 6 | 6 | 0 | -5.110298 | -1.561489 | -0.026039 |
| 7 | 1 | 0 | -4.465168 | 1.779141 | 0.141267 |
| 8 | 1 | 0 | -1.733969 | -1.515254 | -0.342126 |
| 9 | 1 | 0 | -3.641799 | -3.092338 | -0.269406 |
| 10 | 6 | 0 | -1.870193 | 1.255203 | -0.154725 |
| 11 | 6 | 0 | -0.568164 | 1.040126 | -0.392240 |
| 12 | 1 | 0 | -2.171739 | 2.286114 | 0.014999 |
| 13 | 1 | 0 | -0.189815 | 0.040252 | -0.595443 |
| 14 | 6 | 0 | 0.488734 | 2.117468 | -0.366739 |
| 15 | 8 | 0 | -0.139078 | 3.390681 | -0.319691 |
| 16 | 7 | 0 | 1.412584 | 1.993923 | 0.760818 |
| 17 | 1 | 0 | 0.948595 | 1.567103 | 1.557874 |
| 18 | 6 | 0 | 2.732421 | 1.418412 | 0.529535 |
| 19 | 1 | 0 | 3.345512 | 1.619556 | 1.416084 |
| 20 | 1 | 0 | 3.201893 | 1.964585 | -0.297402 |
| 21 | 6 | 0 | 2.786869 | -0.100912 | 0.227674 |
| 22 | 1 | 0 | 2.283939 | -0.637651 | 1.042078 |
| 23 | 1 | 0 | 2.219065 | -0.307234 | -0.687126 |
| 24 | 6 | 0 | 4.205201 | -0.602393 | 0.073895 |
| 25 | 6 | 0 | 4.839212 | -0.591689 | -1.176166 |
| 26 | 6 | 0 | 4.936646 | -1.042270 | 1.185862 |
| 27 | 6 | 0 | 6.163505 | -1.008727 | -1.313041 |
| 28 | 1 | 0 | 4.286198 | -0.259391 | -2.051733 |
| 29 | 6 | 0 | 6.261043 | -1.460395 | 1.054209 |
| 30 | 1 | 0 | 4.459979 | -1.063876 | 2.163219 |
| 31 | 6 | 0 | 6.879508 | -1.444320 | -0.196970 |
| 32 | 1 | 0 | 6.634372 | -0.997672 | -2.291967 |
| 33 | 1 | 0 | 6.808252 | -1.803159 | 1.927847 |
| 34 | 1 | 0 | 7.909293 | -1.772872 | -0.302084 |
| 35 | 8 | 0 | -6.150418 | -2.440017 | 0.015738 |
| 36 | 1 | 0 | -6.962492 | -1.922598 | 0.126660 |
| 37 | 8 | 0 | -6.654959 | 0.182621 | 0.246213 |
| 38 | 1 | 0 | -6.724592 | 1.143537 | 0.303109 |
| 39 | 1 | 0 | 1.104155 | 2.040007 | -1.280544 |
| 40 | 1 | 0 | 0.483016 | 3.943232 | 0.173674 |
